# Scalable Rule-Based Modelling of Allosteric Proteins and Biochemical Networks

**DOI:** 10.1371/journal.pcbi.1000975

**Published:** 2010-11-04

**Authors:** Julien F. Ollivier, Vahid Shahrezaei, Peter S. Swain

**Affiliations:** 1 Centre for Nonlinear Dynamics, Department of Physiology, McGill University, Montreal, Québec, Canada; 2 Centre for Systems Biology at Edinburgh, University of Edinburgh, Edinburgh, United Kingdom; 3 Department of Mathematics, Imperial College London, London, United Kingdom; UT Southwestern Medical Center, United States of America

## Abstract

Much of the complexity of biochemical networks comes from the information-processing abilities of allosteric proteins, be they receptors, ion-channels, signalling molecules or transcription factors. An allosteric protein can be uniquely regulated by each combination of input molecules that it binds. This “regulatory complexity” causes a combinatorial increase in the number of parameters required to fit experimental data as the number of protein interactions increases. It therefore challenges the creation, updating, and re-use of biochemical models. Here, we propose a rule-based modelling framework that exploits the intrinsic modularity of protein structure to address regulatory complexity. Rather than treating proteins as “black boxes”, we model their hierarchical structure and, as conformational changes, internal dynamics. By modelling the regulation of allosteric proteins through these conformational changes, we often decrease the number of parameters required to fit data, and so reduce over-fitting and improve the predictive power of a model. Our method is thermodynamically grounded, imposes detailed balance, and also includes molecular cross-talk and the background activity of enzymes. We use our Allosteric Network Compiler to examine how allostery can facilitate macromolecular assembly and how competitive ligands can change the observed cooperativity of an allosteric protein. We also develop a parsimonious model of G protein-coupled receptors that explains functional selectivity and can predict the rank order of potency of agonists acting through a receptor. Our methodology should provide a basis for scalable, modular and executable modelling of biochemical networks in systems and synthetic biology.

## Author Summary

The complexity of biochemical networks challenges our ability to create quantitative and predictive models of cellular responses to extracellular changes. In these networks, the regulation of allosteric receptors and proteins by multiple drugs or endogenous ligands introduces “regulatory complexity” because a large number of parameters is required to describe such interactions. Protein interactions also give rise to “combinatorial complexity” by generating large numbers of protein complexes and covalent modification states. To address these twin problems, we propose a modelling framework that combines a modular description of protein structure and function with a rule-based description of protein interactions. We define the input-output function of an allosteric protein through its thermodynamic properties and structural components. We show that our “biomolecule-centric” methodology, in contrast to *ad hoc* approaches that emphasize the regulatory logic of interactions, can reduce the number of parameters required to model experimental observations. We also demonstrate how the application of our framework gives insights into the assembly of macromolecular complexes and increases the predictive power of a standard model of G protein-coupled receptors. These benefits are possible in many systems, given the ubiquity of allostery in biochemical networks. Our research delineates a fundamental relationship between allostery, modularity, and complexity in biochemical networks.

## Introduction

A goal of biology is to understand the structure and function of the biochemical networks that underpin cellular decision-making. One organizing principle is that these networks are inherently modular [1]–[3], with specific functions ascribed to a subset of proteins in the network. Yet, like logic gates in electronic circuits, even individual proteins can perform sophisticated computations and integrate multiple inputs [4]–[7]. In engineering, a modular approach to the analysis of a system scales well with the size of the system and its complexity. Indeed, engineers design systems hierarchically with modules comprising other modules. If molecular biology is similarly modular, which structures are the “atomic” modules from which larger modules are constructed? In signalling networks, we may plausibly ascribe this role to protein subunits and domains [8], [9]. Their function as elementary modules often depends on allosteric transitions: an interaction at one site alters the structure at a distant site via a conformational change. Indeed, allostery increases the information-processing ability of a network because it transforms proteins from passive substrates to dynamic computational elements [10]. A modular approach to the analysis and design of biochemical networks should therefore explicitly describe the computations performed by individual allosteric proteins.

Efforts to tackle complexity in biochemical networks should also exploit the modularity of protein structure. Protein structure is hierarchical, and a given protein often has domains also present in other proteins or repeated subunits. For example, many signalling proteins contain SH2 or PDZ domains, and many receptors, ion channels and enzymes are multimers. In genetic networks, transcription factors are also often multimers or have a common DNA-binding domain, such as a zinc finger or homeobox. The re-use of protein domains is both a simplifying and confounding feature: once a domain has been characterized, that characterization can be used again, but it is also necessary to model molecular cross-talk between signalling pathways that contain proteins with similar structures.

*In vivo*, protein interactions can generate both combinatorial and regulatory complexity. Combinatorial complexity is an “explosion” in the number of possible *species* in a system as the number of proteins and interactions in the system increases. It arises because the number of states of a module dramatically increases as its proteins bind ligands as well as each other and as different residues are covalently modified [11], [12]. For example, p53, the so-called cellular “gatekeeper”, has 37 known modification sites and so potentially 2^37^ states [13]. Thus, a complete description of the system potentially requires a combinatorially large number of chemically distinct species and reactions. In contrast, “regulatory complexity” is a combinatorial increase in the number of *parameters* required to describe the regulatory interactions within a system as the number of interactions increase. This complexity arises because the strength of protein interactions depends on the state of a module, and each state of the module potentially requires a unique set of parameters to characterize interactions within the module, with other modules in the network, and with molecules external to the network. Measuring this number of parameters *in vivo* is challenging.

Rule-based modelling addresses combinatorial complexity and allows biologists to specify the regulatory logic of a system [14]. Examples include BioNetGen [15], Kappa [16], Moleculizer [17] and StochSim [18]. Rather than explicitly enumerating each species and reaction in the network, a rule-based model describes a system as a collection of biomolecules interacting according to a set of rules. Each rule is a template for a reaction that specifies the reactants, products and all relevant biochemical parameters. Thus, combinatorially complex systems are compactly described because a large number of distinct reactions are subsumed in the template encoded by a single rule. An algorithm may automatically infer a complete reaction network prior to simulation or, if the combinatorial complexity is too great, use alternative techniques to simulate the system [19], [20]. Importantly, some rules also specify contextual conditions that constrain when an interaction can occur and hence encode the regulatory logic of the network. For example, a rule may allow only a doubly phosphorylated MAP kinase to phosphorylate its substrate.

Rule-based formalisms can describe complex biochemical systems, but inherently offer little guidance on avoiding a number of methodological problems. First, using rules to specify the regulatory logic of a system does not address the system's regulatory complexity. Consider G protein-coupled receptors (GPCRs), which allosterically couple an extracellular ligand-binding site to an intracellular G protein-binding site [21]. GPCRs can be promiscuous, binding multiple intracellular targets [22], [23]. Supposing a given GPCR can bind one of *L* different drugs or endogenous ligands and one of *G* different G proteins, then in principle we require *LG* pair-wise cooperativity parameters to describe how each ligand regulates the GPCR's affinity for each G protein. Thus, the number of regulatory parameters scales with *LG*, and the number of rules also scales with *LG* because each parameter is part of a rule with distinct contextual constraints. Promiscuous allosteric proteins can therefore require a large number of rules and parameters to characterize their interactions.

Second, a module should have a well-described function and be easily re-used and “portable” between systems, but most rule-based formalisms are not inherently modular. Modellers typically treat proteins as “black boxes” and define interactions using biochemical equations. In such “interaction-centric” approaches, the regulation of proteins is encoded by rules with *ad hoc* (system-specific) conditions that no longer apply when the proteins interact with different partners. These *ad hoc* rules obfuscate the mechanism underlying allosteric regulation because they do not show explicitly how the intrinsic structural and thermodynamic properties of allosteric proteins generate their functional properties. In contrast, a “biomolecule-centric” approach would encode regulatory logic in the proteins themselves. Fewer changes to rules would then be required to define how a new set of interaction partners regulates the protein's activity. If a model includes protein domains and subunits, re-use of these components would also be simplified.

Finally, models generated by rule-based methods should be thermodynamically correct. In biochemical networks, there are often sets of reversible reactions that connect into a closed loop, forming a thermodynamic cycle. In many of these cycles no free energy is consumed: for example, when proteins bind multiple ligands, when ligands bind several conformations of a protein, or when ion channels bind multiple agonists and have closed, open, and desensitized states. Thermodynamics imposes a mathematical relationship between the equilibrium constants for all the reactions involved in such cycles: their product must be unity. Equilibrium constants cannot therefore be assigned independently. A thermodynamically correct methodology should ensure that a model satisfies this constraint, ideally by construction.

Here, we present a modular and scalable modelling methodology that alleviates the regulatory as well as the combinatorial complexity of biochemical networks. We first describe our modelling framework, which uses a thermodynamically grounded treatment of allostery in which ligands distinguish only the conformational state of allosteric proteins. We also introduce a rule-based modelling tool that implements our methodology: the Allosteric Network Compiler (ANC). We use ANC to examine how allostery can make macromolecular assembly more efficacious. We then show how our modelling framework describes common mechanisms of allostery by mapping the regulatory properties of a protein onto conformational changes in the protein itself and demonstrate how we can ease the analysis of multiple ligands interacting through an allosteric protein. Next, we discuss how our approach reduces regulatory complexity and thereby increases a model's modularity. Finally, we use our framework to develop a model of G protein-coupled receptors whose regulatory complexity scales with (L+G) instead of LG and consequently has greater predictive power. While our major goal is to introduce a new modular modelling methodology rather than its implementation, we have made ANC and the models we discuss available at: http://swainlab.bio.ed.ac.uk/anc.

## Results

### A structurally and thermodynamically grounded rule-based methodology for modeling allosteric proteins and biochemical networks is implemented in ANC

Our method is based on the Monod-Wyman-Changeux (MWC) paradigm of allostery [24]. We assume that allosteric proteins are dynamic and have one or more structural components, such as domains or subunits, with distinct conformations. These conformational states have different biological activity – for example, a basal state with poor affinity to downstream proteins and an active state that can bind these proteins. Thermal fluctuations cause these allosteric components to transition between their two conformations in either a concerted or sequential fashion [25]. Ligands and other molecules interact non-cooperatively with each conformation of the protein, distinguishing only its conformational state, and so contribute independently to the equilibrium of the allosteric transition. Each such contribution is parameterized by a “regulatory factor” Γ, which gives the fold-change of the equilibrium constant generated by interactions with the ligand. We make a similar independence assumption with respect to the transition state of the allosteric transition such that ligands also contribute independently to the transition's kinetic rate-constants. These contributions to the kinetics are parameterized in terms of one or more “Φ-values”, which give the effect of modifiers on the forward and reverse rates of the allosteric transition (see *Methods*).

Thus, an allosteric protein can be seen as a modular and dynamic computational device, and we can define the input and output of each allosteric component. The input is a “modifier”, a molecule that binds to and locally perturbs the structure of the component; the output is the fraction of time the component spends in each conformation when the allosteric transition is at equilibrium (see *Methods*). Activation corresponds to biasing the equilibrium in favour of the biologically active state; inhibition corresponds to biasing towards the inactive state. Depending on the system, modifiers may be ligands [24], covalent modifications [26], [27], the conformational state of another component [25], [28], [29], or mutations [30], [31].

**Figure 1:**
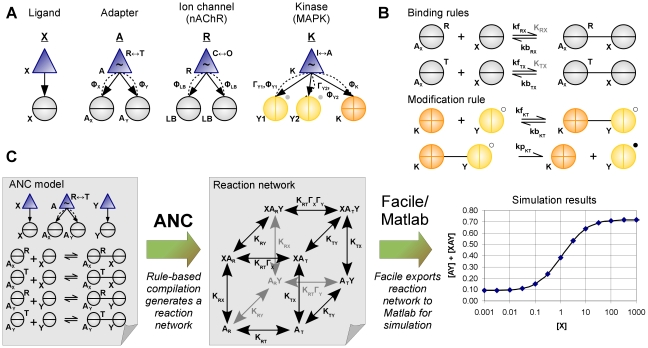
The Allosteric Network Compiler – modelling elements and methodological flowchart. (**A**) Example structures. Each structure has a name (underlined) and comprises a set of named components. Hierarchical components (triangles) represent part or all of a biomolecule and contain, as denoted by arrows, one or more interaction sites (circles). *Left:* The structure X represents a simple ligand with a single binding site (circle with horizontal bar). *Centre-left:* The structure A represents a generic, divalent allosteric adaptor protein. The adaptor's hierarchical component is allosteric (indicated by a tilde) and transitions between low *(R)* and high-affinity *(T)* conformational states. The dashed lines indicate that each binding site acts as a modifier for the allosteric transition, with each interaction parameterized by the indicated Φ-value, and that ligands can distinguish each conformation. *Centre-right:* The structure R is a simplified model of the nicotinic aceltylcholine receptor (nAChR), following Edelstein *et al.* [67] but without desensitized states. The allosteric component transitions between closed *(C)* and open *(O)* states. *Right:* The structure K is a model of a mitogen activated protein kinase (MAPK) with two activating phosphorylation sites (circles with vertical bar and a grey dot as a placeholder for the state) and a catalytic site (circle with cross). The allosteric component transitions between inactive *(I)* and active *(A)* states. Both the phosphorylation sites and the catalytic site are modifiers of the allosteric transition: each successive phosphorylation biases the equilibrium of the enzyme towards the active state by a regulatory factor Γ_Y1_ or Γ_Y2_. Each of these interactions is also parameterized by a distinct Φ-value. (**B**) Example rules. A pair of binding rules for the adaptor A and the ligand X specify the association and dissociation rates of A_X_ with X when A_X_ is in the *R* and *T* states, a similar pair (not shown) specifies the rates for A_Y_ and Y, and we define the affinities K_RX_ and K_TX_ implied by the rates (in gray, e.g. K_RX_ = kf_RX_/kb_RX_). A covalent modification rule for the kinase K acting on an unphosphorylated (open dot) downstream target Y follows the Michaelis-Menten mechanism for enzyme-substrate interactions and yields a phosphorylated substrate (filled dot). (**C**) Methodological flowchart. In a model of the adaptor protein A and its ligands X and Y (Figure 7 of Text S1), the rules state that both ligands bind with higher affinity to the *T* state of the adaptor. This model is compiled by ANC to generate a reaction network where horizontal transitions correspond to conformational changes, vertical transitions correspond to binding the ligand X, and transitions into the page represent binding the ligand Y. K_RT_ is the allosteric equilibrium constant, while the regulatory factors Γ_X_ and Γ_Y_ are the differential affinity of the ligands to each conformation of A and are calculated by ANC using the rate constants given in the rules (e.g. Γ_X_ = K_TX_/K_RX_). The reaction network is converted into ordinary differential equations by *Facile* and these are simulated in *Matlab* to compute the output response of the system (bound A_Y_ vs. X, with A_TOT_ = 1, Y_TOT_ = 1, K_RT_ = 10^−3^ K_RX_ = 0.1, K_TX_ = 10, K_RY_ = 0.01, K_TY_ = 100, arbitrary units).

**Figure 2:**
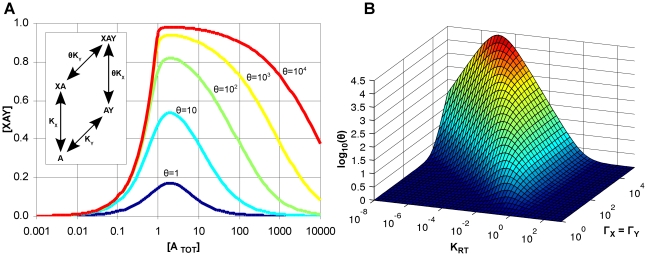
Allostery makes macromolecular assembly robust and controllable. (**A**) Effect of allostery on macromolecular assembly when a linker component is over-expressed. Each curve shows the equilibrium concentration of the XAY trimer against the total amount of A. The total amount of X and Y was unity, while K_RT_ and the affinities of X and Y to each conformation of A (K_RX_, K_RY_, K_TX_, K_TY_) were chosen to yield a desired value of θ and with K_X_ = K_Y_ = 1. *Inset*: A coarse-grained version of the divalent protein model of Figure 1C sums over the two possible conformations of A and shows that with K_X_, K_Y_ and the concentrations of X and Y held constant, the efficacy of assembly depends only on the cooperativity parameter θ. (**B**) Regulation of cooperativity and assembly. The value of θ depends on the other parameters of the model through Equation 1, which is plotted against K_RT_ on one axis and Γ_X_ and Γ_Y_ (assumed equal) on the other. Increasing Γ_X_ and Γ_Y_ always increases cooperativity, however θ has a maximum value as K_RT_ is changed.

**Figure 3:**
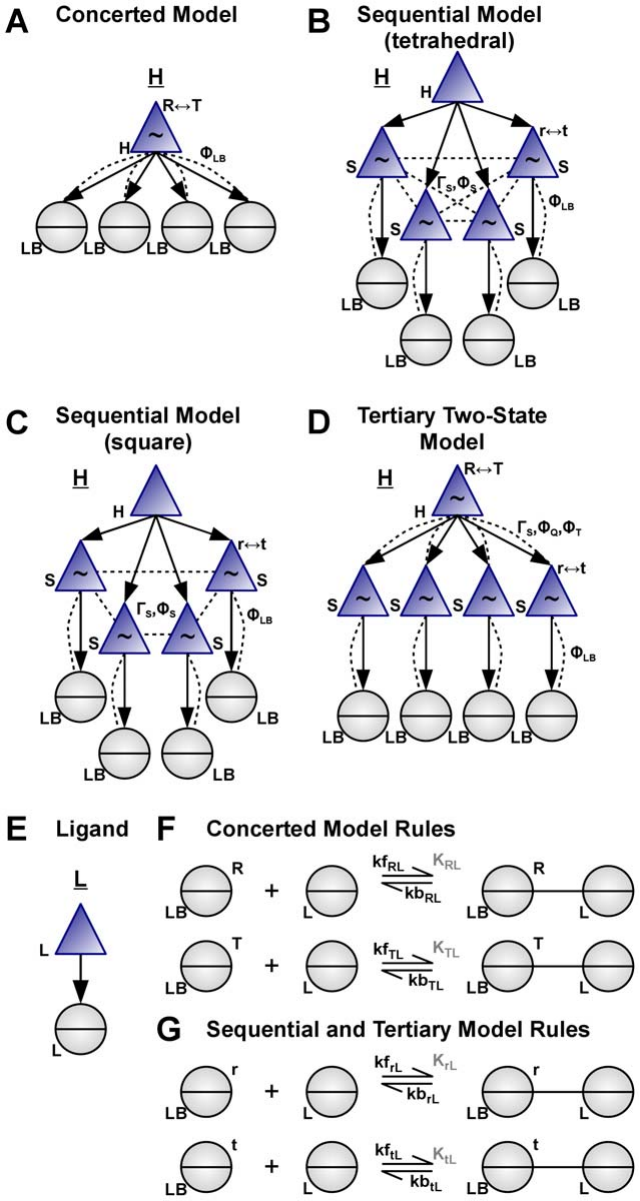
Classic and general models of allostery and protein structure are described by our modelling framework. (**A**) A concerted model of a tetrameric allosteric protein has one allosteric component and 4 identical interaction sites to represent each subunit. The dashed lines indicate that each ligand-binding site is a modifier for the *R*↔*T* allosteric transition and all 4 interactions are identically parameterized by Φ_LB_. (**B**) In a sequential model of the protein, a top-level hierarchical component comprises 4 identical allosteric components that individually change conformation and bind ligand. These components are allosterically coupled (dashed lines) such that each subunit is equivalent and a modifier for all neighbouring subunits – the “tetrahedral” model. The strength of the coupling is given by the regulatory factor Γ_S_ and the effect of each modifier on the kinetics of coupled components is parametrized by Φ_LB_ and Φ_S_. (**C**) Altered lateral interactions between subunits gives the “square” model. (**D**) A tertiary two-state model has one allosteric hierarchical component containing 4 identical allosteric components, each with a ligand-binding site. The upper quaternary component is allosterically coupled to each tertiary component with strength Γ and the tertiary components are coupled to their binding site. The effect of the quaternary conformation on the kinetics of the tertiary transition is given by Φ_Q_, and the reciprocal interaction is parameterized by Φ_T_. (**E**) The ligand for all four models. (**F**) Rules for the concerted model in panel A. (**G**) Rules for the models in panels B, C and D.

**Figure 4:**
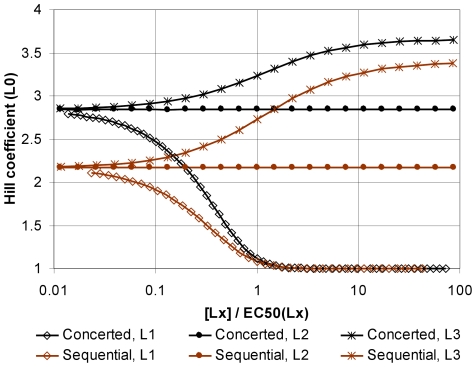
Cooperative binding of competitive ligands to the concerted and sequential models. The allosteric equilibrium of an unligated protein favours a state *T* (or *t*). Ligand L0 binds preferentially to state *R* (or *r*) and so binds cooperatively to the protein. The Hill coefficient of the dose-response function for L0 (the number of L0 bound to the protein versus the concentration of L0) was measured in the presence of increasing concentrations of three competing ligands: L1 favours the *R* state; L2 is neutral; L3 favours the *T* state. Concentrations of competing ligands are normalized to the EC50 of their own occupancy function. For the concerted model K_RT_ = 10^3^; for the sequential (tetrahedral) model K_rt_ = 0.1 and Γ_S_ = 10. Ligand affinities were set to K_RLi_ = K_rLi_ = (Γ_i_)^−1/2^ and K_TLi_ = K_tLi_ = (Γ_i_)^1/2^ with Γ_0_ = Γ_1_ = 0.01 (prefers *R* or *r*), Γ_2_ = 1 and Γ_3_ = 100 (prefers *T* or *t*).

**Figure 5:**
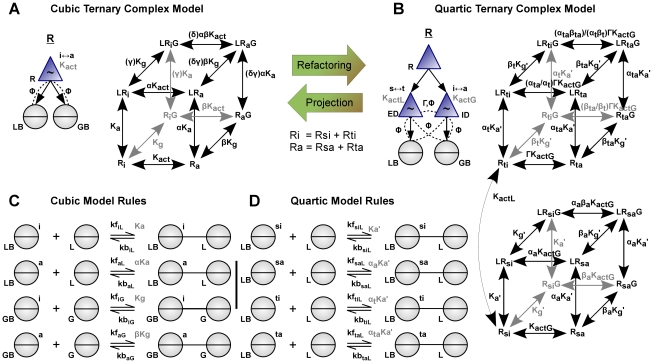
Cubic and quartic ternary complex models of a GPCR in our modelling framework. The mapping between the cubic (A) and quartic (B) models shows how the two models are related. (**A**) A naive implementation of the cubic ternary complex model. The ANC-structure R has one allosteric component which transitions between a low-affinity, inactive (*i*) state and a high-affinity, active (*a*) state with the indicated equilibrium constant (in gray). LB and GB are binding sites for an extracellular ligand L (not shown) and an intracellular target G protein (not shown). In the corresponding cubic, 8-state transition diagram K_act_ is the unligated allosteric equilibrium constant, K_a_ and K_g_ are ligand affinities to the reference (inactive) state, and α and β are ratios of affinities. We parenthesize the cooperativity parameters δ and γ to indicate that these parameters of the cubic ternary complex model have to be added as *ad hoc* rules to the naïve implementation. (**B**) In our quartic ternary complex model, an ANC-structure R comprises two allosteric components: the extracellular domain ED transitions between low and high-affinity states (*s* and *t*); the intracellular domain ID transitions between inactive and active states (*i* and *a*). These transitions are reciprocally linked (dashed line) so each domain acts a modifier of the other with the interaction parameterized by Γ and Φ. The binding sites are allosterically coupled to *both* allosteric components, therefore each ligand “sees” 4 possible conformations of the receptor. In the quartic state-transition diagram K_actG_ and K_actL_ are the unligated allosteric equilibrium constants, Г is the regulatory factor linking the *s*↔*t* and *i*↔*a* transitions, K_a_′ and K_g_′ are ligand affinities to the reference state *si*, and α and β are ratios of ligand affinities of the subscripted state relative to the reference state. For clarity, we show only the unligated *s*↔*t* transition. (**C**) Rules for the cubic ternary complex model showing the rate and equilibrium constants for ligand and G protein binding. (**D**) A subset of the rules for the quartic ternary complex model shows the rate and equilibrium constants for ligand binding. A similar set of rules specifies rate and equilibrium constants for binding G protein (Figure 9 of Text S1).

**Figure 6:**
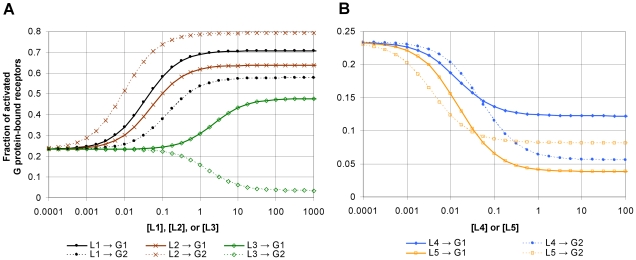
Functional selectivity of agonists in the quartic ternary complex model. (**A**, **B**) We simulated the GPCR-mediated (in)activation two target G proteins by several ligands. A dose-response for each ligand and G protein pair shows the amount of receptor species capable of signalling (R_sa_G+R_ta_G+LR_sa_G+LR_ta_G) as a fraction of the total number of receptors and against the concentration of ligand (arbitrary units). The concentrations of receptor and G protein are unity. Parameter values: KactL = 1, KactG = 0.05, Γ = 1, affinities for L1 are given by: (Ka′, α_t_, α_a_, α_at_) = (10,0.1,10,1), for L2: (1,20,20,400), L3: (0.1,10,10,0.01), L4: (100,0.1,0.4,0.01) L5: (20,20,0.05,5), G1: (Kg′, β_t_, β_a_, β_at_) = (10,0.1,10,1) and G2: (1,10,10,100).

An ANC model consists of a set of modular structures and interaction rules. Using our rule-based approach (Tables 1–5 of Text S1, Figures 1–9 of Text S1) and building on the thermodynamic framework described in *Methods*, each molecule in a system is described using an ANC construct called a *structure*, which captures the true structure of a protein in terms of its components (Figure 1A). ANC-structures contain two types of components: hierarchical components and interaction sites. Hierarchical components have two roles. The first is that of containment and composition: a hierarchical component typically contains interaction sites but can also contain other hierarchical components. Hierarchical components may represent a unit of tertiary structure, such as a protein domain, or of quaternary structure, for example, a protein with multiple subunits. Their second role is to undergo conformational transitions if designated as allosteric, following the two-state model described in *Methods*. Interaction sites are of three types: catalytic sites (such as a kinase or phosphatase), sites that can be covalently modified, and ligand-binding sites. Next, *rules* specify the interactions between sites and how the strength of these interactions depends on the conformational state or the covalent modifications of a protein (Figure 1B). If the interaction is a binding reaction, the rule gives the association and dissociation rates. If the interaction is enzymatic – such as a phosphorylation or dephosphorylation – then we assume a Michaelis-Menten mechanism, and the rules give the rate of formation of the product and the association and dissociation rates between the sites, which must be a catalytic site and a covalent modification site.

The overall modelling process for a divalent adaptor protein and two ligands is illustrated in Figure 1C. Structures and rules are entered as text and saved to a file (section §2.7 of Text S1). ANC reads the file, creates an initial set of seed structures, and launches an iterative compilation algorithm. At each iteration, the algorithm determines all inter- and intramolecular reactions, the products created, and their biochemical rates. In a subsequent iteration, the newly created products may in turn react to produce yet more species. Once a final biochemical reaction network has been obtained, it is simulated using deterministic or stochastic methods (see *Methods*). The deterministic simulation in Figure 1C shows how upon binding X, which could represent an activated receptor, the adaptor A recruits increasing amounts of Y, which could represent a downstream signalling protein.

### Analysis of the cooperativity of ligand binding to a generic divalent allosteric protein

The generic model of a divalent allosteric protein shown in Figure 1C (full details in Figure 7 of Text S1) can be used to model proteins that play other roles than adaptors. For example, A could be a membrane-bound receptor, X an extra-cellular agonist, and Y an intracellular signalling protein which binds preferentially to the active conformation of the receptor. The usefulness and simplicity of this model motivates us to analyze it mathematically.

In the model, the binding of X and Y to A is cooperative because binding of X to A changes the affinity of A for Y by a factor θ and likewise the binding of Y to A changes the affinity of A for X also by a factor θ. By coarse-graining over the conformations of A (Figure 2A, inset), we can express the cooperativity parameter θ as (section §2.1 of Text S1):
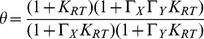
(1)where K_RT_ is the allosteric equilibrium constant and Γ_X_ (or Γ_Y_) is the differential affinity of the X (or Y) to each conformation of A. The cooperativity increases as the degree of bias (Γ_X_ and Γ_Y_) that X and Y exert on the conformational transitions of A increase. We can also define the apparent affinity of X and Y to this coarse-grained A:
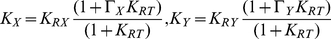
(2)These equations relate the underlying parameters of the model to experimental observables: both the affinity K_X_ of X to A and the affinity θK_X_ of X to A when A is bound by Y are measurable.

### Allostery can make macromolecular assembly robust even when linker proteins are over-abundant

Counter-intuitively, an excess of some components of a macromolecular complex can inhibit formation of the complex [32], [33]. This phenomenon, called the prozone effect, is strongest for a protein that links two or more separable parts of a complex. It occurs because the linker protein competes with itself for the binding of the other components of the complex, and so if it is present in excessively high amounts, few of the linker proteins will succeed in simultaneously binding all the other components, resulting in partially formed complexes.

Here, we show that allostery can mitigate the prozone effect, at least for a divalent allosteric protein. We consider the divalent structure A to represent a linking protein with X and Y being the remaining parts of a complex. In Figure 2A, we demonstrate how increasing the cooperativity increases the range of concentrations of A for which assembly of the complex XAY is efficacious (i.e. where the equilibrium amount of complex exceeds 50% of the maximum amount). This range increases from 1.3 decades when θ = 1 to 4.0 decades when θ = 10^4^, and the maximal amount of complex formed increases by a factor of 5.7 (Figure 11 of Text S1). Thus, an allosteric linker protein has a dual benefit in macromolecular assembly: it both increases the amount of complex when the components are present in their stochiometric amounts and makes complex formation more robust to the over-expression of the linker protein. Allosteric linker proteins could explain the low correlation observed between over-expression of a linker protein and lethality of that over-expression in budding yeast [34].

That the efficacy of macromolecular assembly depends strongly on the value of the cooperativity parameter θ suggests that assembly could be modulated by changing θ. Figure 2B shows the dependence of θ on the allosteric equilibrium constant and the differential affinity of the ligands X and Y. Cooperativity has a maximum at K_RT_ = (Γ_X_Γ_Y_)^−1/2^ and thus assembly of the XAY trimer could in principle be controlled through the binding of a cofactor or a covalent modification that changes the allosteric equilibrium constant of A from a value far from its optimum to a value near the optimum (or vice versa).

### A compact, structural and modular representation of allosteric proteins

There are two well-known mechanisms for generating cooperative behaviour in proteins: concerted and sequential allostery. In their seminal paper, Monod, Wyman and Changeux introduced a two-state model to explain cooperative interactions in oligomeric enzymes and proteins [24]. They proposed that all subunits of such proteins undergo a *concerted*, reversible, and quaternary-level transition between two conformational states. Ligand-binding to each conformation is non-cooperative, but each conformation differs in its affinity for ligands and this difference gives rise to cooperative effects. Subsequently, Koshland, Nemethy and Filmer lifted the assumption of concerted transitions with their *sequential* model, in which each subunit transitions individually between two conformational states [25]. This model can explain negative cooperativity in oligomeric proteins. It assumes, however, that ligands cause an instantaneous conformational change, or an *induced fit*, in the structure of the subunit. Both the concerted and induced fit assumptions can be relaxed and are special cases of the general allosteric model of Herzfeld and Stanley [28].

ANC-structures can be used to implement these models of allosteric regulation. A concerted model of a generic, homotetrameric protein is shown in Figure 3A. The transition between the two conformations, labelled *R* and *T*, is concerted because a single allosteric component contains all subunits. Cooperativity will arise if a ligand has a higher affinity for one state, say the *R* state, and if the unligated protein is mostly in the alternate state *T*. Then, once bound by ligand, the protein spends more time in the *R* state and so favours the binding of additional ligands. In contrast, a sequential model has an ANC-structure with four allosteric components, each with *r* and *t* states. Figures 3B and 3C show how we implement the tetrahedral and square geometries described by Koshland *et al.* [25] through different configurations of the allosteric coupling between subunits. Two components are allosterically coupled if the conformation of one component biases the conformational equilibrium of the other component, and vice versa. For example, in Figures 3B and 3C, ligand binding favours the *r* state of an individual subunit, and this subunit when in its *r* state favours the *r* state in those subunits to which it is allosterically coupled and so generates a cooperative response. Finally, we illustrate the general approach with the tertiary two-state model shown in Figure 3D, which allows both quaternary, *R*↔*T* and tertiary, *r*↔*t*, allosteric transitions [29]. Here, ligand binding favours *r* at the bound subunit because of the ligand's higher affinity for *r*. Cooperativity arises because the *R* state of the quaternary structure reciprocally favours the *r* state of the tertiary subunits. Thus, a subunit in the *r* state favours *R* at the quaternary level and so favours subunits not yet bound by ligand to also be in the *r* state, promoting binding of additional ligands. Our ANC implementation of the tertiary two-state model correctly reproduced the Henry *et al.* model, which has 252 molecular species [29].

An advantage of ANC is its ability to easily formulate and simulate mathematically complex models. For example, we will show that the cooperativity of an allosteric protein binding a ligand, such as a transcription factor binding an inducer, can be substantially changed through adding a competing ligand. Although a mathematical analysis of various allosteric models with two competing ligands exists [35], little is known about multiple ligands and the analysis is cumbersome for the sequential model despite simplifying assumptions. Using ANC, we characterized the binding cooperativity of a ligand L0 to a tetrameric allosteric protein in the presence of one of three different competing ligands for both the concerted and sequential models (Figure 4). In the absence of competitors, the Hill coefficients for binding L0 in the concerted and sequential models were ∼2.8 and ∼2.2 respectively. By increasing the concentration of the competitor ligand L1, which binds preferentially to the same conformation as L0, the Hill coefficient decreased progressively to 1 (i.e. no cooperativity). With ligand L3, which binds preferentially to the low affinity state for L0, the Hill coefficients increased to ∼3.6 and ∼3.4. With ligand L2, which binds with equal affinity to all conformations, the Hill coefficient did not change. With competitors L2 and L3, the EC50 of L0 binding increased but at low concentrations of L1, the EC50 of L0 was slightly lower (Figure 12 of Text S1).

In addition to ligand binding, our methodology also describes other mechanisms for allosteric regulation that are ubiquitous in cellular signalling. Phosphorylation or other post-translational modifications, dimerization, receptor clustering and point mutations can also regulate or change protein function. Our thermodynamic framework (see *Methods*) unifies the treatment of such heterogeneous modifiers of protein activity. In section §2.4 of Text S1, we discuss how dimerization and ligand binding jointly regulate the activity of the epidermal growth factor receptor, and how ligand binding combines with methylation to regulate a chemotaxis receptor (Figure 13 of Text S1).

### Encoding regulatory logic through intensive rather than extensive parameters reduces regulatory complexity and increases modularity

We can distinguish two types of parameters that affect modularity in different ways: *intensive* parameters and *extensive* parameters. Intensive parameters describe the conformational transitions and intramolecular interactions of a protein and, as such, are modular because they are inherent to the protein and independent of the protein's interaction partners. Therefore, we associate intensive parameters with a protein's ANC-structure. In contrast, extensive parameters describe the interactions of a protein with other biomolecules and increase in number as the number of these interactions increase. Extensive parameters, contained in rules for interaction, are the “wiring” between modules and are non-modular because they depend on the system in which the protein functions. Regulatory complexity occurs when the number of extensive parameters describing a system scales combinatorially with the number of interactions in the system.

Our biomolecule-centric methodology minimizes regulatory complexity. For example, we analyzed a generic model of an *N*-valent, two-state protein where each of the *N* binding sites is unique and binds exactly *L* ligands (section §2.5 and Table 6 of Text S1). In an “interaction-centric” modelling approach, there are no intensive parameters and the number of extensive parameters scales as *L^N^*. In the “biomolecule-centric” methodology of ANC, there are 2 intensive parameters and the number of extensive parameters scales linearly with the number of interactions *NL*. Using our methodology can therefore yield large savings; for instance if N = 6 and L = 5, we have 91 independent rate constants rather than over 233,000 (Table 7 of Text S1). Thus, by encoding the regulatory logic of proteins with intensive rather than extensive parameters, we reduce regulatory complexity. We therefore improve the model's modularity because only extensive parameters change when a model is updated.

### Refactoring yields a scalable and modular GPCR model that can explain functional selectivity

Using our biomolecule-centric modelling framework, we can convert a non-modular model into a modular one. Such refactoring is also useful when a protein has more than two conformational states, unlike the core allosteric components in ANC-structures. To illustrate, we introduce a new model for the activation of G protein-coupled receptors (GPCRs). GPCRs are a common target for pharmaceutical drugs [36]. Such drugs include agonists that promote activation of the receptor, inverse agonists that promote deactivation of the receptor, and antagonists that by binding to the receptor block the action of agonists.

Although several allosteric models have been proposed [37], [38], we will consider the cubic ternary complex model [39] because this model describes the constitutive activity of a GPCR, the action of inverse agonists and antagonists, and how some inverse agonists can cause a GPCR to recruit G proteins but remain inactive [40]. The model also explains the functional selectivity of receptors (also called agonist trafficking, ligand-biased agonism, or protean agonism) [41] through the notion of active states of the receptor that are specific to a ligand or a G protein [38], [42]. However, the model does not include receptor homo- or hetero-oligomerization [43]–[48], or the possibility that GPCRs form stable, pre-assembled complexes with downstream proteins [49], [50].

A naive implementation of the cubic ternary complex model in our framework uses a divalent ANC-structure with a single allosteric component (Figure 5A, C). This implementation captures Weiss *et al.*'s assumption that the receptor has only two conformational states. However, it does not capture the cooperative binding of a ligand to either the inactive or active states of the receptor because such binding is incompatible with the paradigm that each modifier contributes independently to the equilibrium constant of the allosteric transition [24]. This cooperative effect is described in Weiss *et al.*'s model through the cooperativity parameters γ and δ. However, these parameters are extensive and specific to each combination of ligand and G protein. They therefore introduce regulatory complexity.

To resolve this difficulty, we propose a sequential allosteric model of the GPCR with two coupled allosteric components: an extracellular allosteric component, which binds a ligand, and an intracellular allosteric component, which binds a G protein (Figure 5B, D). The ligand and the G protein interact simultaneously with *both* allosteric components. They therefore “see” four possible conformations of the receptor instead of two. These conformations are implied in the cubic ternary complex model because each ligand has four distinct affinities to the receptor. However, none of the extensive parameters in our model are cooperativity parameters specific to a ligand-G protein pair, thus eliminating regulatory complexity.

Our quartic ternary complex model can be projected onto the cubic model by defining coarse-grained variables that sum over the conformations of the extracellular allosteric component (Figure 5 and section §2.6 of Text S1). The “inactive” and “active” states in the cubic model therefore correspond to a mixture of conformational states, providing a mechanism for how different ligands induce an apparently unique conformation of the activated GPCR with a distinct affinity for the G protein [51]. In our model, each ligand uniquely affects the allosteric equilibrium of the extracellular domain and therefore the fraction of time that the receptor is in the *s* and *t* states, which in turn uniquely modulates the affinity of the active GPCR for the G protein.

Our quartic model for the GPCR is more modular and parsimonious than the cubic model because it includes a structurally and biophysically plausible mechanism for how ligands and G proteins interact cooperatively with the GPCR. We encode the logic of these regulatory interactions in the protein's ANC-structure using intensive parameters, rather than in *ad hoc* rules with extensive parameters. Our “refactored” model has 11 parameters (3 of which are intensive) compared to the 7 parameters of the cubic model (1 of which is intensive) and double the number of states. This initial cost for increased modularity and “portability” becomes a benefit as the number of types of ligands and G proteins increases. The number of extensive parameters in our model scales linearly with the number of interactions; in the cubic model, the number of extensive parameters scales combinatorially. For example, suppose we wish to model 4 different ligands that activate the thyroid-stimulating hormone receptor. In human thyroid membranes, this GPCR can activate at least 10 different G proteins [22]. With 4 ligands and 10 G proteins, our quartic model is almost twice as parsimonious as the cubic model, requiring 59 rather than 109 parameters.

The quartic model also has more predictive power than the cubic model and therefore can be more rigorously tested. For each pair of ligands and G proteins, the cubic model requires the specification of two cooperativity parameters, δ and γ, specific to that pair. It is therefore limited in the predictions it can make. For example, for each new G protein added to the system, new cooperativity parameters are needed for all previously characterized ligands to be able to predict the new G protein's GPCR-mediated response to these ligands. In contrast, the quartic model is completely characterized for the new target pathway by measuring four extensive parameters – one for each conformation of the GPCR – and we can then predict the GPCR-mediated response to all ligands. In particular, we can predict the rank order of potency of the ligands to activate the new pathway, a standard means to compare agonists in pharmacology, and detect functional selectivity [51].

Like the cubic model, the quartic ternary complex model also explains functional selectivity, though this is not obvious considering that these models cannot be related through a simple projection when multiple ligands and G proteins interact with a single receptor. Indeed, in the quartic model δ and γ are not free parameters but are correlated because of their dependence on underlying rates. We therefore simulated the GPCR-mediated response to several ligands that cause (in)activation of two different G proteins (Figure 6A and 6B). Ligand L1 has the greatest ability to activate G protein G1 as measured by its potency (−log(EC50) of the response) and efficacy (maximal activation). For G1, ligand L2 has intermediate potency and efficacy and L3 has the lowest potency and efficacy. Also, L1 is better able to activate G1 than G2. If the receptor had only a single active state, we would therefore infer that this state must have a poorer ability to bind and activate G2 and would expect that L2 should also have a decreased ability to activate G2. The potency and efficacy of L2 on G2 is, however, greater than that of L1 indicating a reversal in the rank order of the potency and efficacy of L1 and L2. Also, ligand L3 is an agonist for G1 but an inverse agonist for G2. These observations of agonists selectively (in)activating distinct target pathways cannot be reconciled with a model comprising a single active state of the receptor. Functional selectivity can also be observed for the repression of activity by inverse agonists because there are also two *inactive* states of the receptor (Figure 6B).

The quartic model is modular and therefore is easily extended to include additional signalling interactions such as the regulation of the receptor by allosteric ligands [52], [38]. Also, by adding dimerization sites, we could incorporate existing models of dimerization of GPCRs [47]. Some GPCRs may also oligomerize *in vivo*, for instance by forming tetramers [53]. We could model oligomerization by concatenating multiple receptor models within a larger ANC-structure through modular composition. A starting point could be one of the models of Figure 3, but substituting for each subunit the ANC-structure for a GPCR and, as appropriate, adding allosteric couplings to model inter-receptor interactions.

## Discussion

Biochemical networks are complex yet modular: networks exhibit both combinatorial and regulatory complexity, but individual proteins have intrinsic functional properties that determine how they detect and process information. Complexity is also reduced because similar proteins or similar protein domains appear in many signalling pathways and often interact with similar protein partners. We propose a modelling methodology, embodied by ANC, that exploits the modularity of proteins to reduce the complexity of modelling biochemical networks. Given modular ANC-structures, which encode a protein's regulatory properties, adding new interactions to an ANC model usually requires substantially fewer parameters than with other rule-based models, particularly as the promiscuity of binding of proteins, and hence the complexity of the network, increases. ANC-structures are also portable because different signalling pathways are modeled by simply “re-wiring” proteins rather than through writing new *ad hoc* rules encoding the regulatory logic specific to each pathway.

In our methodology, models are structured to minimize regulatory complexity both to avoid over-fitting data and because large numbers of biochemical parameters are difficult to measure *in vivo*. Indeed, our modelling framework reflects a natural division between two classes of parameters: “intensive” parameters describe the allosteric transitions and intramolecular interactions of a particular protein and are attributes of ANC-structures; “extensive” parameters describe the interactions of the protein with other biomolecules and are associated with rules. In different biochemical networks, only the extensive parameters of a protein change. Through its assumption that a ligand or substrate distinguishes only the conformation of a protein or enzyme and not its occupancy or state of covalent modification at distant sites, our modelling framework substantially reduces the number of extensive parameters required: their number scales linearly, rather than combinatorially, with the number of interactions. This reduction in extensive parameters allows a model for the interaction of a protein with individual ligands to also predict the response to mixtures of ligands with no additional parameters [54]. We have shown that interference with a competing ligand can either increase or decrease the cooperativity of the response to the original ligand (Figure 4) – a potentially useful mechanism of control.

Our methodology reduces the regulatory complexity but increases the combinatorial complexity of a system because each conformation of an allosteric protein introduces a new state. Thus, a reduction in regulatory complexity incurs the computational cost of modelling additional species. Nevertheless, recent advances in rule-based modeling have introduced new methods that allow fast simulation of systems with large numbers of chemical species and reactions [17], [19], [20], [55]–[57]. By focusing on avoiding an exponential increase in extensive parameters in systems with promiscuous binding, our methodology both complements and potentially benefits from these innovations.

We also make a first step at integrating free energy-based constraints into a rule-based modelling framework, adding to earlier work on imposing detailed balance in models of biochemical networks [58]–[61]. By automatically computing all dependent extensive parameters associated with allosteric transitions – the allosteric equilibrium and rate constants for each ligated and modified state – from the appropriate independent parameters, ANC prevents the modeller from incorrectly specifying these parameters. Thus, cycles comprising allosteric transitions are biophysically correct by construction. For complex models with a combinatorially large number of occupancy states and covalent modifications, this automation is essential.

Two other advantages of our modelling framework are significant. First, ANC-structures enable a coarse-grained hierarchical description of physical structure by requiring the specification of protein domains and if desired tertiary and quaternary structure, including oligomeric receptor clusters. ANC-structures can also model the internal geometry of a protein by describing those domains of the protein that interact allosterically and those that do not (Figure 3). Second, the thermodynamic framework underpinning our method offers a systematic and unified way to model how proteins integrate heterogeneous inputs such as ligands, phosphorylations, or even small mutations to compute an output response.

Our modelling framework encourages the modeller to develop a mechanism to explain the regulatory properties of a protein and hence to build models that have predictive power and so can be experimentally tested. For example, an ANC model of the activation of GPCRs suggests that the well-known cubic ternary complex model has implicitly coarse-grained some conformations of the GPCR. By including these conformations in an ANC-structure, our new quartic model prevents over-fitting and has the potential to predict the rank order of potency and efficacy of ligands acting through a GPCR. This model of the GPCR has two linked allosteric components, each with just two conformational states that interact independently with other molecules. These mechanistic assumptions do not, however, apply to the GPCR as a whole, which has four conformational states. Thus, while the two-state assumption may not hold for all proteins, other mechanistic models can be accommodated within our framework.

Having allostery at its centre, our framework can suggest simple mechanisms through which the cell might regulate and increase the efficacy of cellular processes. For example, the assembly of macromolecular complexes can be considerably undermined through the prozone effect when linker proteins are over-expressed [32]. Consequently we might expect that expression of the components of macromolecular complexes is tightly regulated. Such regulation can be complex and expensive. Yet modelling with our framework suggests that if allosteric proteins are part of the macromolecular complex and if the linker proteins are allosteric then the prozone effect can be substantially reduced and without energy input (Figure 2A).

A challenge in designing synthetic biological systems is to have predictive modelling tools. Here, ANC has several potential advantages. First, the modularity of ANC-structures allows models of synthetic systems to be straightforwardly extended: for example, as different synthetic subsystems are combined to generate more complex behaviour [7]. Second, through its rule-based modeling and the specification of rules of interaction between protein components rather than between proteins, ANC naturally models molecular cross-talk between synthetic sub-circuits in a larger synthetic circuit and between a synthetic circuit and the endogenous biochemistry (if a rule-based model of endogenous signalling is available). In both cases, ANC will find and model interactions if proteins are present that happen to have complementary binding domains. Such interactions could, for example, affect the formation of macromolecular signalling complexes (Figure 2) or change the Hill coefficient of the response of a crucial pathway (Figure 4). Finally, an ANC model includes background activity in all enzymes because control of each enzyme is described by an allosteric transition between inactive and active conformations. This transition will occur regardless of the presence of input signals to the system, although the probability of such occurrences can be small. Like molecular cross-talk, background activity can cause a synthetic circuit to deviate substantially from its designed behaviour.

Faced with the complexity of cellular signalling and genetic networks, researchers are developing new computational methods to quantitatively model and predict cellular behaviour despite that complexity. In this spirit, we have identified and discussed a distinct form of complexity – regulatory complexity – which arises from the allosteric regulation of proteins. Combining and extending established biophysical principles with more recent rule-based methods, we propose a modular and scalable methodology, exemplified by our Allosteric Network Compiler, to describe the complexity of cellular signalling. By emphasizing the allosteric control of proteins, we capture the inherent modularity of protein structure and function exploited by cells themselves. Our method is a general, principled and simplifying addition to any modeling framework.

## Methods

### Thermodynamic framework

To compute how multiple modifiers collectively bias the conformational equilibrium of an allosteric component, we use thermodynamics [62]. We arbitrarily designate one conformation as the reference state *R* and the alternate conformation as *T*. If we let 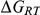 be the difference in free energy between the *R* and the *T* conformations in the absence of any modifiers, then the difference in free energy in the presence of *N* modifiers is, quite generally [63],
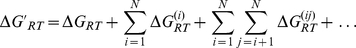
(3)where we include contributions of free energy to the new allosteric equilibrium that are determined by each modifier alone, by pair-wise interactions between modifiers, and by all higher order interactions.

We assume that all modifiers interact independently (non-cooperatively) with each conformational state of the protein component, with the energy of interaction to the *R* state of the component given by 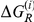 and to the *T* state by 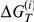 for the modifier indexed by *i*. Consequently, the free energy required to apply a modifier to each conformation does not depend on the presence or absence of other modifiers – a modifier can only distinguish the conformational state of the component. Therefore we need consider just the first order terms of equation (1) and ignore higher order interactions:
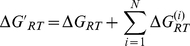
(4)For each modifier, a reversible thermodynamic cycle exists around which the change in free energy must be zero. For example, equilibria exist between the *R* and the *T* states of the component, between the modifier *i* being applied to the *R* state (a free energy change of 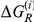), the modifier being applied to the *T* state (a free energy change of 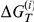), and between the *R* and the *T* states of the modified form of the component. To have no change in free energy around this cycle implies that 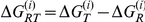. Hence, we have:
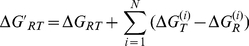
(5)From statistical mechanics, we know that the equilibrium constant between any two states of a system, say A and B, is connected to the difference in their free energy through the expression
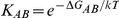
Therefore, we may exponentiate equation (3) to find the corresponding equilibrium constant:
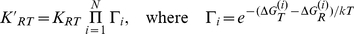
(6)with *kT* denoting the product of Boltzmann's constant and temperature.

Equation (6) describes the input-output function of an allosteric component, which may embody a domain, a subunit, or an entire protein. The output 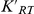 is the allosteric constant of the component under the effect of N modifiers. It is obtained by multiplying a baseline equilibrium constant 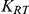 with each “regulatory factor” Γ*_i_*, which describes the effect of an input modifier *i* on the allosteric equilibrium. If the modifier is a ligand, then Γ*_i_* is the ratio of the ligand's affinity to each conformation. If the modifier is a covalent modification such as a phosphorylation, the regulatory factor is an independent parameter related to the free energies required to phosphorylate each conformation. If the modifier is another allosteric component to which the component is allosterically coupled (e.g. Figure 3B–D), then Γ*_i_* is an independent parameter related to the free energy of interaction of the *T* form of the modifier with each conformation of the allosteric component, and gives the fold-change in the allosteric equilibrium constant induced by the *T* form of the modifier. When this modifier is in its reference state, which we label *R*, the output is by definition unchanged and the regulatory factor is not applied.

To compute how the kinetics of a component's allosteric transition are affected by the presence of modifiers, we first write the forward and backward rate constants for the unmodified component in terms of the free energy difference between the transition state (denoted †) and each conformational state [64]:
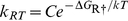
(7a)

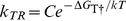
(7b)To obtain the rate constants for a modified state, the simplest approach is to assume a constant pre-exponential factor *C* and that modifiers contribute independently to a change in the free energy of the transition state (section §1.2 of Text S1). The assumption of independence is not arbitrary: at the core of the MWC paradigm of allostery is the assumption that modifiers contribute independently to the free energy of each conformational state. Here we extend this idea to the transition state of the allosteric transition. As a result, modifiers independently affect the kinetic rates, just as they do the equilibrium constant, and we can write:
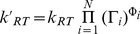
(8a)

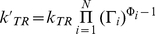
(8b)for parameters Φ_i_.

We choose this parameterization because Φ_i_ = Φ_j_ implies the existence of a linear free energy relationship for two modifiers *i* and *j* (section §1.3 of Text S1). A linear free energy relationship [65] is a common, simplifying assumption in biophysical models: in a set of related reactions, the logarithm of a transition rate is assumed to be linearly related to the logarithm of the equilibrium constant [31], [66]. The parameter Φ denotes the proportionality constant. Assuming a linear free energy relationship to model simultaneous modifiers, for example in models of hemoglobin or of the nicotinic acetylcholine receptor [29], [66], [67], also implies that these modifiers independently affect the conformational transition, with each effect parameterized by the same value of Φ (section §1.3 of Text S1).

### Validation, testing, modelling and simulation

We validated our overall methodological flow (Figure 1C) and verified the output of ANC by implementing and simulating an allosteric model [68] of the signalling protein calmodulin (Figure 10A and Figure 10B of Text S1). Binding of calcium to calmodulin modulates its affinity for downstream effectors. We confirmed that ANC correctly generates the 352 biochemical equations of the model of Stefan *et al.* and that our simulation results were consistent with theirs, using their experimentally derived parameter values (Figure 10C of Text S1).

ANC possesses a number of features which ease modelling and simulation of biochemical networks. First, ANC allows users to parameterize a model so that parameter values can be changed after compilation. Also, ANC supports *stimuli*, through which the user can apply input waveforms to specified nodes in the network, and *probes* – user-defined collections of molecules – to measure network output. Finally, ANC allows the creation of *ad hoc* regulatory conditions to support interaction-centric approaches. Such *ad hoc* conditions, however, reduce the modularity and scalability of a model and so do not play to the strength of our methodology.

Using Facile [69], an application distributed with ANC, we can export an ANC-compiled network to standard tools such as Matlab, XPP, Maple or Mathematica for deterministic simulation or analysis, to EasyStoch for stochastic simulation [70], or to SBML [71].

### Limitations of method

The current implementation of ANC has three principle limitations. 1) The reaction network is enumerated, so ANC's performance may degrade significantly if the compiled network is large. 2) Only rules for binding and Michealis-Menten interactions can be created. 3) While ANC supports unimolecular association and dissociation, detailed balance is enforced only for cycles comprising purely bimolecular associations.

## Supporting Information

Text S1 Supplementary information. This file contains all supplementary information for the article.
(4.94 MB PDF)
