# Supplementary material for: Scalable Rule-Based Modelling of Allosteric Proteins and Biochemical Networks
Source: PLoS Comput Biol. 2010 Nov 4;6(11):e1000975. doi: 10.1371/journal.pcbi.1000975 (PMC2973810; doi:10.1371/journal.pcbi.1000975)
Supplement: Text S1 — Supplementary information. This file contains all supplementary information for the article. (4.94 MB PDF) [file pcbi.1000975.s001.pdf]

SUPPLEMENTARY INFORMATION

This document provides supplementary information for the article:

Ollivier JF, Shahrezaei V, Swain PS. "Scalable rule-based modeling of allosteric proteins and biochemical networks", PLoS Comp. Bio., 2010.

TABLE OF CONTENTS

1 Supplementary Methods ... 2
1.1 Rule-based compilation of allosteric models ... 2
1.1.1 Constituents of an ANC model ... 2
1.1.1.1 Components ... 2
1.1.1.2 Structures ... 4
1.1.1.3 Structure Instances ... 6
1.1.1.4 Initial Conditions ... 6
1.1.1.5 Rules ... 7
1.1.2 Biochemical network generation ... 8
1.1.2.1 Generation of a binding reaction ... 8
1.1.2.2 Generation of a covalent modification reaction ... 9
1.1.2.3 Generation of allosteric transitions ... 10
1.1.3 Examples of network generation ... 11
1.1.3.1 Model of a divalent adaptor protein ... 12
1.1.3.2 Concerted allosteric model of a tetramer ... 14
1.1.3.3 Quartic ternary complex model of a G protein-coupled receptor ... 17
1.2 Derivation of kinetic input-output function ... 21
1.3 The Φ parameter, linear free energy relationships, and independence ... 22
1.4 Validation of ANC using a model of calmodulin ... 24
2 Supplementary Results ... 25
2.1 Mathematical analysis of a generic divalent allosteric protein and two ligands ... 25
2.2 Effect of allosteric cooperativity on the width and maximum response of XAY trimer assembly ... 26
2.3 Effect of competitive ligands on the EC50 of ligand in the concerted and sequential models ... 27
2.4 Examples of the allosteric regulation of proteins and receptors by heterogeneous mechanisms ... 28
2.5 Regulatory complexity ... 30
2.5.1 Equilibrium analysis ... 30
2.5.1.1 Ad hoc approach ... 30
2.5.1.2 ANC approach ... 31
2.5.2 Including kinetic rates ... 33
2.5.2.1 Ad hoc approach ... 33
2.5.2.2 ANC approach ... 33
2.5.3 Discussion of regulatory complexity ... 34
2.5.4 Summary of regulatory complexity analysis ... 35
2.5.5 Comparison of interaction-centric and biomolecule-centric approaches ... 36
2.6 Derivation of QTC to CTC mapping functions ... 37
2.7 ANC Model of Adaptor Protein ... 38
3 Supplementary References ... 42

1 Supplementary Methods

1.1 Rule-based compilation of allosteric models

The aim of this section is to specify ANC's rule-based modelling framework in sufficient detail so that the reader can infer the set of biochemical equations and rates which ANC generates given a model.

1.1.1 Constituents of an ANC model

An ANC model comprises a set of components, a set of structures, a set of rules, and a set of initial conditions. Auxiliary modelling constructs also allow the user to specify input waveforms, network readouts, and various options. These auxiliary constructs are fully discussed in the ANC User Manual available online (http://swainlab.ed.ac.uk/anc).

1.1.1.1 Components

An ANC model comprises a set of named, typed and re-usable components which are used to build structures. A component's type determines its role within a structure and which class of biochemical reactions apply to it. Table 1 lists each type of component, its graphical symbol and associated attributes.

Table 1: Component types and attributes.

Type	Graphical symbol	Attributes	Notes
Interaction sites			
Binding site	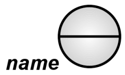	*name*	Participates in reversible binding reactions. The binding site's name must distinguish it from other interaction sites.
Catalytic site	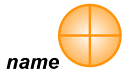	*name*	Acts as the catalytic site in enzyme reactions. The catalytic site's name must distinguish it from other interaction sites.
Modification site	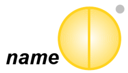	*name*	Acts as the substrate in enzyme reactions. The modification site's name must distinguish it from other interaction sites.
Hierarchical components			
Hierarchical component	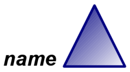	*name*	A modular element, is used to "contain" other components in a structure, and can be composed to create modular structures. Each hierarchical site has a unique name to distinguish it from other hierarchical or allosteric components.
Allosteric component	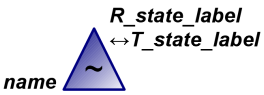	*name,*<br>*R_state_label,*<br>*T_state_label,*<br>*k_RT,*<br>*k_TR*	Dual role as a hierarchical and allosteric component capable of adopting one of two allosteric states. Each allosteric component has a unique name to distinguish it from other hierarchical or allosteric components. The other attributes, which may optionally be included in the graphical symbol, are labels for the reference state and the non-reference state (defaulting to *R* and *T*), and baseline allosteric transition rate constants.

1.1.1.2 Structures

An ANC model comprises a set of named structures. As shown in the example of Figure 1, an ANC-structure is a named, labelled and partially directed graph comprising a set of nodes and edges linking the nodes. Each node is associated with a component, whose graphical symbol is used when drawing the structure. Nodes are labelled, and therefore distinguishable, by the name and type of their associated components. Through the association of each node with a component, a structure models the relationship between the components of biomolecules (this association also allows us to use the terms "node" and "component" interchangeably in most contexts). Edges, which may be directed, are labelled and distinguishable according to the edge type and (for allosteric couplings) interaction parameters. Table 2 describes each type of edge used to build a structure. Nodes and edges are not necessarily unique – indeed, multiple nodes may be associated with the same component to model, for example, identical binding sites or subunits.

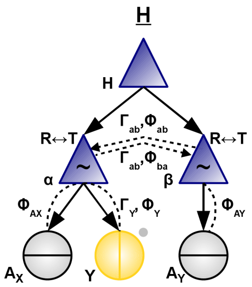

Figure 1: Example structure. This structure models a hypothetical protein H. The structure's name is underlined to distinguish it from the names of its components. Protein H has two allosteric domains, α and β, undergoing sequential transitions. The modifiers of domain α are a ligand binding site A_X, a phosphorylation site Y, and the conformational state of domain β. Domain β is regulated by a binding site A_Y and by the state of the domain α. The allosteric coupling between α and β subunits consists of two directed edges because, while thermodynamics imposes that the regulatory factor Γ_{ab} is the same in both directions, the Φ-values characterizing the effect of each subunit's conformational state on the other's transition kinetics can be different.

Table 2: Structure edges.

Type	Graphical symbol	Label	Notes
Edges			
Containment edge	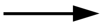	None	A containment edge can only be drawn from a hierarchical (or allosteric) node, but can point to any other node type.
Binding edge		None	A binding edge can only be drawn between two interaction sites, and represents a non-covalent bond between these sites.
Allosteric coupling edge (from modifier to allosteric node).	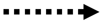	Φ or Γ, Φ	When drawn pointing to an allosteric node, means that the other node acts as a modifier of the allosteric transition with the indicated Φ-value and (if appropriate) regulatory factor Γ. If this modifier is an interaction site, the label comprises only a Φ-value, since in this case Γ is not a static value but depends on the differential affinity of the ligand occupying the binding site. If the modifier is a modification site or another allosteric component, then the edge is labelled with both Γ and Φ.
Allosteric coupling edge (from allosteric node to interaction site).	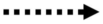	None	When drawn from an allosteric node to an interaction site, means that the interaction site can “see” the conformational state of the allosteric node.
Allosteric coupling			Given that allosteric couplings necessarily come in pairs, for convenience each pair of directed edges may be drawn as a single undirected edge, as we have done in the main text and elsewhere.

1.1.1.3 Structure Instances

A user-defined structure can be *instantiated* to create a *structure instance*. A structure instance is a copy of the instantiated structure to which state information is annotated. As illustrated in Figure 2, this annotation consists of appending relevant state information (if any) to the label of each component-associated node.

Structure instances are created when a model is initialized with initial conditions prior to compilation. During compilation, new structures and structure instances are dynamically created as needed to represent products of the biochemical reactions implied by the model. Generally speaking, structures embody the static, non-changing attributes of a biomolecule which are common to all its instances, while instances capture state information that may change with time and as a result of a reaction.

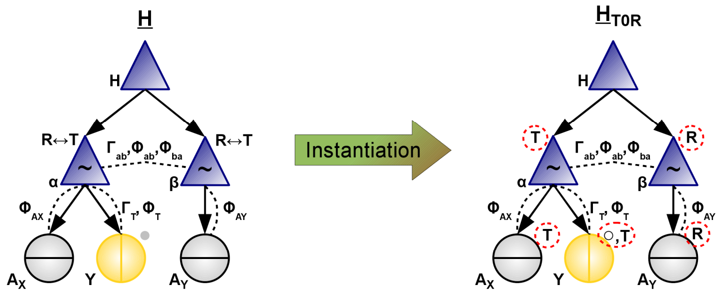

Figure 2 Structure instantiation. A structure instance named H_{T0R} of structure H is created by copying the structure H and incorporating relevant state information (dotted red circles) to the labelling of each node. During instantiation, the state of each allosteric node becomes one of the allosteric state labels defined by the user (in this case, R or T). The state of modification sites becomes either 0 (open circle) or 1 (filled circle). All interaction sites also inherit the conformational state of any allosteric nodes to which they are coupled. For simplicity, we have drawn the pair of directed allosteric coupling edges connecting α and β as a single undirected edge, despite the ambiguity of which Φ-value "points to" which subunit.

1.1.1.4 Initial Conditions

An ANC model also comprises a set of initial conditions. Each initial condition specifies the following information:

- i) the name of a structure
- ii) the state of each component in the structure and
- iii) the initial concentration of the instance (representing a particular chemical species).

During initialization of the model, initial conditions are used to *instantiate* the associated structures, creating an initial set of seed structure instances to which reaction rules are iteratively applied to generate a reaction network. The initial concentration specified does not affect network generation but does affect simulation results.

1.1.1.5 Rules

An ANC model comprises a set of rules which are created (either explicitly or implicitly) from three pre-defined templates, namely a binding rule template, a covalent modification rule template, and an allosteric transition rule template (Figure 3). Thus, an ANC model contains the information required to create *instances* of the rule templates. Each rule instance is a copy of a rule template but specifies additional information such as the name and state of the components involved and rate constants.

Instances of binding and modification rules (Figure 3A and 3B) are explicitly created by the modeller using ANC language constructs. Each binding or covalent modification rule instance comprises an association rule instance and a dissociation rule instance. Additionally, the covalent modification rule instance comprises a product rule instance. These 3 types of *elementary rule instances* are used as generators to create binding and enzymatic reactions in a biochemical reaction network.

In contrast to the explicit creation of binding rule instances, an instance of the allosteric transition rule template (Figure 3C) is automatically created for each allosteric component in a model, without the modeller explicitly requesting it. Each allosteric transition rule instance comprises an elementary rule instance for the transition from the component's reference state to its non-reference state, and a second elementary rule instance for the opposite transition. These elementary rule instances are used as generators to create allosteric transitions in a reaction network.

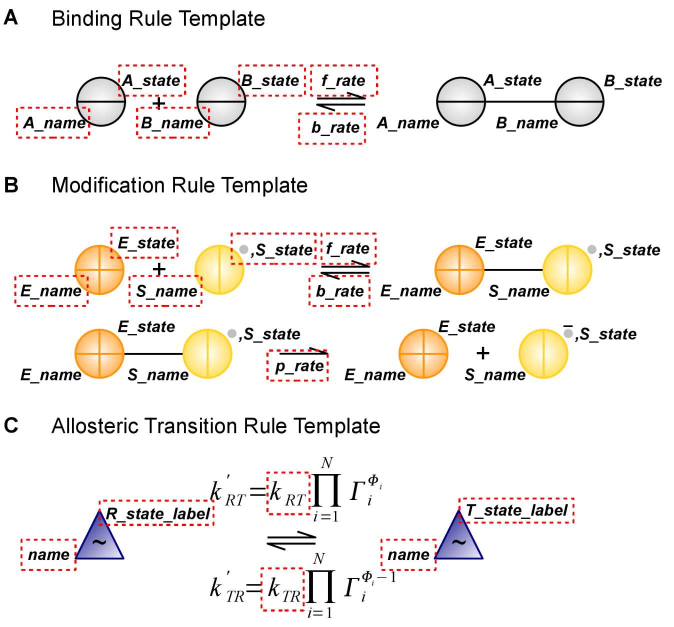

Figure 3: Binding, covalent modification, and allosteric transition rule templates. Rule instances are created from one of three pre-defined templates: a binding rule template, a modification rule template, and an allosteric transition template. **(A)** The binding rule template comprises two elementary rules. To create binding rule instances, the modeller supplies the information required by the template (dotted red boxes): the name of each interaction site and biochemical rate constants. The modeller may also, if desired, specify the state of each interaction site: either the modification state, the conformational state or both. **(B)** The covalent modification rule template is similar to the binding rule template but comprises a third elementary rule corresponding to the product reaction of the Michaelis-Menten mechanism. The product rule specifies that the modification state of the substrate *S_name* (represented by a grey dot) is flipped in the product of the reaction (grey dot with an overbar). To create modification rules instances the modeller supplies interaction site names, interaction site states, and biochemical rates. Specifying the state of the substrate site prior to modification defines what type of enzyme is involved (e.g. if 0, a kinase and if 1, a phosphatase). **(C)** The allosteric transition rule template comprises elementary rules for the transition to and from the reference conformational state. The template includes a built-in formula for calculating allosteric transition rates given that N modifiers are present in a particular case. ANC automatically creates an instance of the allosteric transition rule template for each allosteric component in a model, obtaining from the component's attributes both labels for the reference and non-reference states (by default, *R* and *T*) and baseline allosteric transition rates.

1.1.2 *Biochemical network generation*

ANC's iterative algorithm uses elementary rule instances to generate a biochemical reaction network. To do so, the algorithm matches the left-hand side (LHS) of each elementary rule against all the structure instances in the network. A match is conditioned by a component's type, name and state as specified in a rule, but regardless of which structure contains the components of the LHS (though additional *ad hoc* matching conditions can be specified – c.f. the ANC User Manual). A rule instance may match a structure instance multiple times if the structure contains multiple copies of a component matched by the rule's LHS. Each distinct match generates a biochemical reaction and new structures and structure instances are created to represent the products of the reaction (such as a complex of two structures), as appropriate. In a subsequent iteration, the compilation algorithm can match the rules against the newly created products, compiling new biochemical reactions until a stopping condition is reached.

1.1.2.1 *Generation of a binding reaction*

Figure 4 illustrates how a binding rule instance is used to generate a reversible binding reaction.

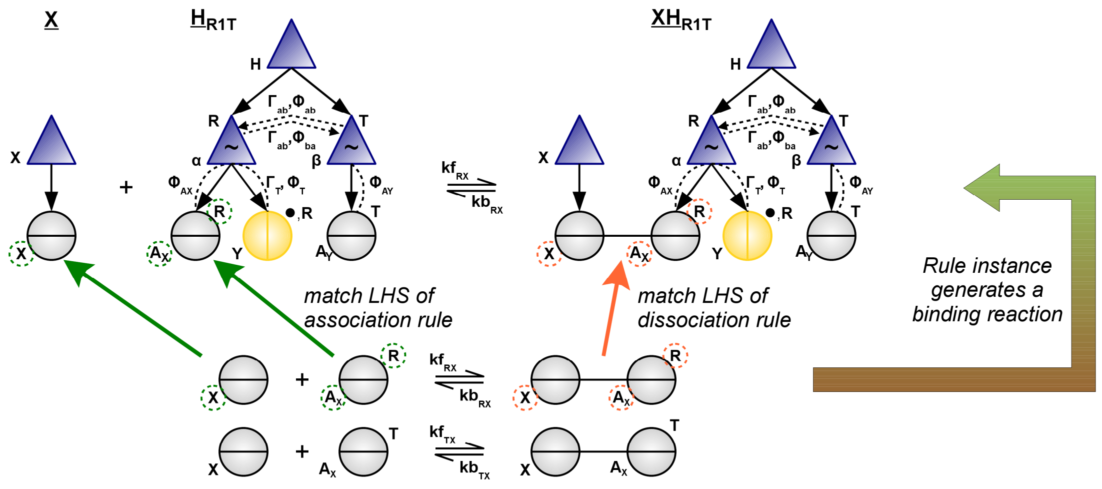

Figure 4: Using a binding rule instance to generate a reversible binding reaction. We suppose that the reaction network already contains structure instances X and H_{R1T} representing a ligand and a protein. As a pair, these structures are checked against the rule instances in the model to see whether a binding reaction can be generated. Two binding rule instances exist for the reversible binding of interaction sites X and A_X. However, only one of them correctly matches the allosteric state of A_X (green dotted circles and arrows). The elementary rule instance for association guides the construction of a new structure instance representing the protein-ligand complex, and generates a bi-molecular association reaction with the rate constant kf_{RX}. After comparing the new structure instance with those already existing in the reaction network to avoid unnecessary duplications, the new product structure is assigned a unique name XH_{R1T}. At a later time, the compilation algorithm matches the complex against the elementary dissociation rule instance (orange dotted circle and arrow), and generates the dissociation reaction with the rate kb_{RX} specified by the rule.

1.1.2.2 Generation of a covalent modification reaction

Figure 5 illustrates how a modification rule instance is used to generate a Michaelis-Menten reaction.

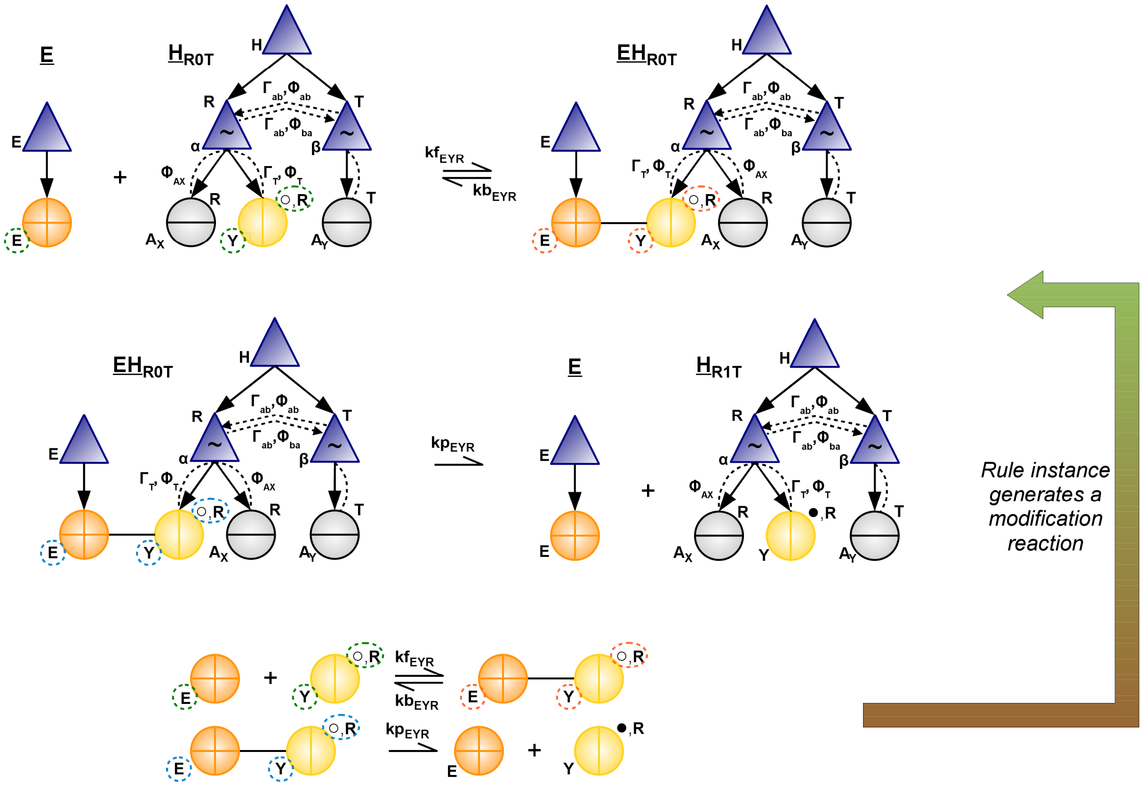

Figure 5: Using a modification rule instance to generate an enzymatic reaction. We suppose that the reaction network already contains structure instances E and H_{R0T} representing a kinase and a protein having a phosphorylation site Y. As a pair, these structures are checked against the rule instances in the model to see whether a modification reaction can be generated. The interaction sites E and Y (the latter in an unmodified state and *R* conformation) match the association rule instance comprised by the modification rule (green dotted circles). The elementary rule instance for association guides the construction of a new structure instance representing the enzyme-substrate complex, and generates a bi-molecular association reaction with the rate constant kf_{EYR}. After comparing the new structure instance with those already existing in the reaction network to avoid unnecessary duplications, the new product structure is assigned a unique name EH_{R0T}. Next, the compilation algorithm matches the complex against the elementary dissociation rule instance (orange dotted circles), and generates the dissociation reaction with the rate kb_{ERX} specified by the rule. Finally, the compilation algorithm also matches the enzyme-substrate complex against the elementary product rule instance (blue dotted circles) and generates a product reaction with rate kp_{ERX}. After checking that the structure instance doesn't already exist in the network, the phosphorylated product is assigned the name H_{1RT}.

1.1.2.3 *Generation of allosteric transitions*

Figure 6 illustrates how the allosteric transitions of the α subunit of protein H are generated when the protein is bound to ligand X and given the state of the other components of the protein.

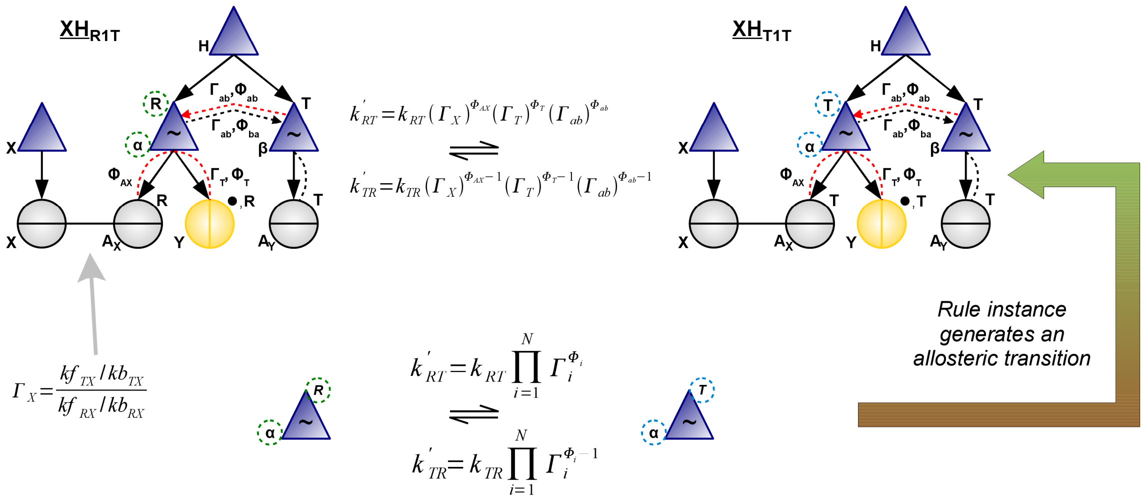

Figure 6: Using an allosteric transition rule instance to generate an allosteric reaction. We suppose that the reaction network already contains the structure instance for the ligand-protein complex XH_{R1T}, whose α subunit is in state *R*. The name and state of the component matches the LHS of the elementary allosteric transition rule (dotted green circles) and so the compilation algorithm generates an allosteric transition to the opposite state. The algorithm creates a new structure instance and, assuming it doesn't already exist in the network, assigns it the name XH_{T1T}. To compute the transition rate constant k_{RT}', the baseline rate of the transition k_{RT} is multiplied by a factor corresponding to each modifier affecting the transition (red dashed lines). In this particular case, there are N=3 modifiers since the interaction site Y is modified (black dot), the β subunit is in its non-reference conformational state *T*, and since the binding site A_X is occupied by ligand X. The regulatory factors and Φ-values are obtained from the labelling of the allosteric coupling edges of the modifiers, except in the case of the ligand X for which the regulatory factor Γ_X is the differential affinity of the ligand X to each conformational state. As shown, Γ_X is calculated from the rate constants of the binding rules shown in Figure 4. Subsequently, the compiler matches the new structure instance XH_{T1T} against the *T*→*R* elementary rule (blue dotted circles), creates the reverse allosteric transition and calculates the rate constant k_{TR}' according to the prescribed formula.

1.1.3 Examples of network generation

The aim of this section is to demonstrate, through some concrete examples, how the rule-based framework described above is used to generate a model's reaction network. In each example, a figure gives the structures and rules of the model (which the modeller creates in a textual form using ANC language constructs), and a diagram of the reaction network implied by the model. Also, each example has a table that lists every reaction generated by the compilation algorithm, the reaction's rate constant, and the rule instance that generated it. References to each rule instance specify which elementary rule was used: for binding rules f=association, b=dissociation; for allosteric transitions f=transition from reference state, b=transition to reference state. Allosteric transition rate constants are calculated according to the prescription of the template with the indicated number of modifiers.

Note that to improve legibility, the names of the structure instances given here (e.g. XA_TY) may differ from the names actually generated by ANC. Also, the specific order in which reactions are listed (and in which new structures are generated) may not be identical to the order in which the compilation algorithm generates them, both for clarity and because future updates and improvements to the implementation of the algorithm may change this order. However, each complete reaction network listed here is identical to that generated by ANC.

1.1.3.1 Model of a divalent adaptor protein

The first example is a model of generic, divalent adaptor protein A interacting with two ligands X and Y. As shown in Figure 7A and 7B, the model comprises three structures and 4 binding rule instances. Table 3 shows how the compilation algorithm applies these rules to create the reaction network shown in Figure 7C.

Note that this model is isomorphic to the “naïve” form of the cubic ternary complex model of a GPCR shown in Figure 5 of the main text.

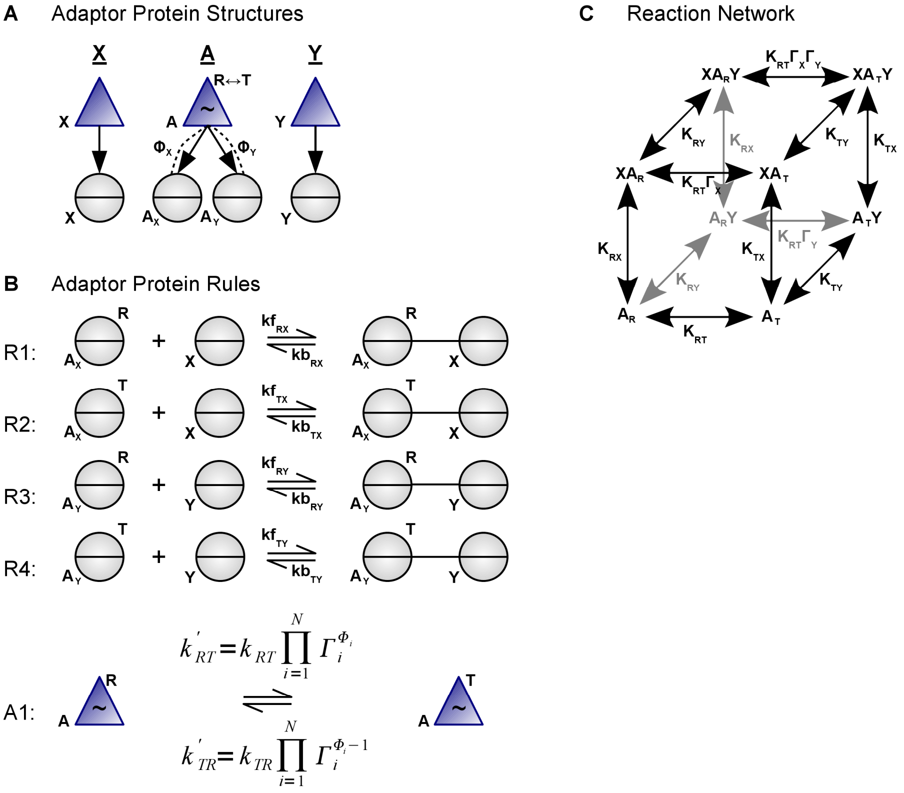

Figure 7: ANC model of a generic, divalent adaptor protein. A) The model comprises 3 structures named X, A and Y, which are instantiated with initial conditions (not shown). **B)** The binding rule instances R1-R4 are explicitly defined in the model. The allosteric transition rule instance A1 is automatically generated by ANC for the allosteric component. **C)** After application of the rules, a biochemical reaction network arises which we represent as a cube whose vertices correspond to the 8 possible states of the adaptor protein. The edges represent reversible transitions and we have annotated the equilibrium constants $K_{RT} = k_{RT} / k_{TR}$, $K_{RX} = kf_{RX} / kb_{RX}$, $K_{TX} = kf_{TX} / kb_{TX}$, $K_{RY} = kf_{RY} / kb_{RY}$, $K_{TY} = kf_{TY} / kb_{TY}$ and the regulatory factors $\Gamma_X = K_{TX} / K_{RX}$ and $\Gamma_Y = K_{TY} / K_{RY}$.

Table 3: Network generation for a model of a generic, divalent adaptor protein.

This table illustrates how the rule instances in Figure 7 are used by the iterative compilation algorithm to generate binding and allosteric reactions, using the rate constants associated with each rule instance, and creating new structure instances as needed.

**Rule applied**	**Reaction**	**Rate constant**	**New species added to network**	**Notes**
Initialization			A_R, X, Y	We assume the model defines initial conditions for these structure instances (i.e. species).
A1(f)	$A_R \rightarrow A_T$	k_{RT}	A_T	A is unligated, so there are no modifiers (N=0).
A1(b)	$A_T \rightarrow A_R$	k_{TR}	–	A is unligated, so there are no modifiers (N=0).
R1(f)	$A_R+X \rightarrow XA_R$	kf_{RX}	XA_R	Association of A_R and X creates complex XA_R.
R1(b)	$XA_R \rightarrow A_R+X$	kb_{RX}	–	Dissociation of A_R and X.
R2(f)	$A_T+X \rightarrow XA_T$	kf_{TX}	XA_T	Association of A_T and X creates complex XA_T.
R2(b)	$XA_T \rightarrow A_T+X$	kb_{TX}	–	Dissociation of A_T and X.
R3(f)	$A_R+Y \rightarrow A_RY$	kf_{RY}	A_RY	Association of A_R and Y creates complex A_RY.
R3(b)	$A_RY \rightarrow A_R+Y$	kb_{RY}	–	Dissociation of A_R and Y.
R4(f)	$A_T+Y \rightarrow A_TY$	kf_{TY}	A_TY	Association of A_T and Y creates complex A_TY.
R4(b)	$A_TY \rightarrow A_T+Y$	kb_{TY}	–	Dissociation of A_T and Y.
R1(f)	$A_RY+X \rightarrow XA_RY$	kf_{RX}	XA_RY	Association of A_RY and X creates complex XA_RY.
R1(b)	$XA_RY \rightarrow A_RY+X$	kb_{RX}	–	Dissociation of A_RY and X.
R2(f)	$A_TY+X \rightarrow XA_TY$	kf_{TX}	XA_TY	Association of A_TY and X creates complex XA_TY.
R2(b)	$XA_TY \rightarrow A_TY+X$	kb_{TX}	–	Dissociation of A_TY and X.
R3(f)	$XA_R+Y \rightarrow XA_RY$	kf_{RY}	–	Association of XA_R and Y creates complex XA_RY.
R3(b)	$XA_RY \rightarrow XA_R+Y$	kb_{RY}	–	Dissociation of XA_R and Y.
R4(f)	$XA_T+Y \rightarrow XA_TY$	kf_{TY}	–	Association of XA_T and Y creates complex XA_TY.
R4(b)	$XA_TY \rightarrow XA_T+Y$	kb_{TY}	–	Dissociation of XA_T and Y.
A1(f)	$XA_R \rightarrow XA_T$	$k_{RT}(\Gamma_X)^{\Phi_X}$	–	N=1 and $\Gamma_X = (kf_{TX} / kb_{TX})/(kf_{RX} / kb_{RX})$.
A1(b)	$XA_T \rightarrow XA_R$	$k_{TR}(\Gamma_X)^{\Phi_X - 1}$	–	
A1(f)	$A_RY \rightarrow A_TY$	$k_{RT}(\Gamma_Y)^{\Phi_Y}$	–	N=1 and $\Gamma_Y = (kf_{TY} / kb_{TY})/(kf_{RY} / kb_{RY})$.
A1(b)	$A_TY \rightarrow A_RY$	$k_{TR}(\Gamma_Y)^{\Phi_Y - 1}$	–	
A1(f)	$XA_RY \rightarrow XA_TY$	$k_{RT}(\Gamma_X)^{\Phi_X}(\Gamma_Y)^{\Phi_Y}$	–	N=2.
A1(b)	$XA_TY \rightarrow XA_RY$	$k_{TR}(\Gamma_X)^{\Phi_X - 1}(\Gamma_Y)^{\Phi_Y - 1}$	–	

1.1.3.2 Concerted allosteric model of a tetramer

This example is a model of a tetrameric protein H with a 4-fold axis of symmetry that undergoes concerted allosteric transitions and binds ligand L through 4 identical binding sites. As shown in Figure 8A and 8B, the model comprises two structures and 2 binding rule instances. Table 4 explains how the compilation algorithm applies these rules to create the reaction network shown in Figure 8C.

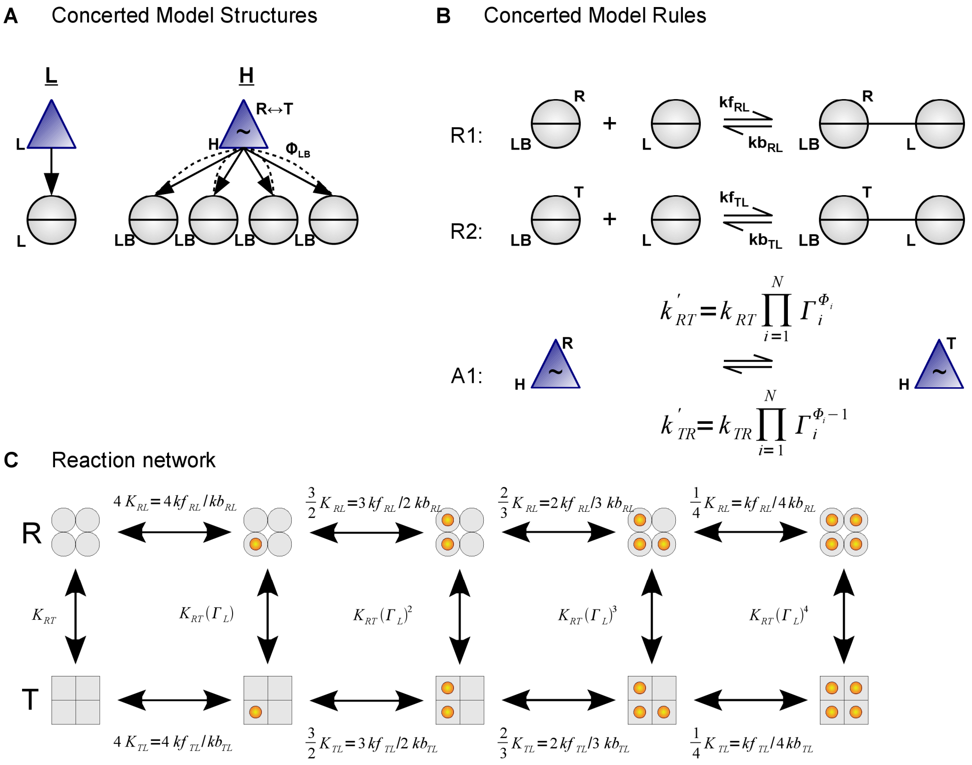

Figure 8: Concerted allosteric model of a tetrameric protein. A) The model comprises 2 structures named L and H, which are instantiated with initial conditions (not shown). **B)** The binding rule instances R1-R2 are explicitly defined in the model. The allosteric transition rule instance A1 is automatically generated by ANC for the allosteric component H. **C)** Through the application of the rules, a biochemical reaction network is generated in which 5 occupancy states exist for each conformation of the protein. The affinity of each conformation of the tetramer to the ligand changes according to how many binding sites are occupied, and the allosteric equilibrium constant is also a function of the occupancy state. However, the affinity of a particular subunit remains independent of the occupancy state. Edges represent reversible transitions and we have annotated the equilibrium constants $K_{RT} = k_{RT} / k_{TR}$, $K_{RL} = kf_{RL} / kb_{RL}$, $K_{TL} = kf_{TL} / kb_{TL}$, and the regulatory factor $\Gamma_L = K_{TL} / K_{RL}$.

Table 4: Network generation for a concerted allosteric model of a tetramer.

This table illustrates how the rule instances in Figure 8 are used by the iterative compilation algorithm to generate binding and allosteric reactions, using the rate constants associated with each rule instance, and creating new structure instances as needed.

**Rule applied**	**Reaction**	**Rate constant**	**New species added to network**	**Notes**
Initialization			H_R, L	We assume the model defines initial conditions for these structure instances (i.e. species).
A1(f)	$H_R \rightarrow H_T$	k_{RT}	H_T	H is unligated, so there are no modifiers (N=0).
A1(b)	$H_T \rightarrow H_R$	k_{TR}	–	H is unligated, so there are no modifiers (N=0).
R1(f)	$H_R+L \rightarrow H_RL$	kf_{RL}	H_RL	Association of H_R and L creates H_RL.
R1(f)	$H_R+L \rightarrow H_RL$	kf_{RL}		2nd match for association rule.
R1(f)	$H_R+L \rightarrow H_RL$	kf_{RL}		3rd match for association rule.
R1(f)	$H_R+L \rightarrow H_RL$	kf_{RL}		4th match for association rule.
R1(b)	$H_RL \rightarrow H_R+L$	kb_{RL}	–	Dissociation of H_R and L.
R2(f)	$H_T+L \rightarrow H_TL$	kf_{TL}	H_TL	Association of H_T and L creates H_TL.
R2(f)	$H_T+L \rightarrow H_TL$	kf_{TL}		2nd match for association rule.
R2(f)	$H_T+L \rightarrow H_TL$	kf_{TL}		3rd match for association rule.
R2(f)	$H_T+L \rightarrow H_TL$	kf_{TL}		4th match for association rule.
R2(b)	$H_TL \rightarrow H_T+L$	kb_{TL}	–	Dissociation of H_T and L.
R1(f)	$H_RL+L \rightarrow H_RL_2$	kf_{RL}	H_RL_2	Association of H_R L and L creates H_RL_2.
R1(f)	$H_RL+L \rightarrow H_RL_2$	kf_{RL}		2nd match for association rule.
R1(f)	$H_RL+L \rightarrow H_RL_2$	kf_{RL}		3rd match for association rule.
R1(b)	$H_RL_2 \rightarrow H_RL+L$	kb_{RL}	–	Dissociation of H_R and L.
R1(b)	$H_RL_2 \rightarrow H_RL+L$	kb_{RL}	–	2nd match for dissociation rule.
R2(f)	$H_TL+L \rightarrow H_TL_2$	kf_{TL}	H_TL_2	Association of H_T L and L creates H_TL_2.
R2(f)	$H_TL+L \rightarrow H_TL_2$	kf_{TL}		2nd match for association rule.
R2(f)	$H_TL+L \rightarrow H_TL_2$	kf_{TL}		3rd match for association rule.
R2(b)	$H_TL_2 \rightarrow H_TL+L$	kb_{TL}	–	Dissociation of H_T and L.
R2(b)	$H_TL_2 \rightarrow H_TL+L$	kb_{TL}	–	2nd match for dissociation rule.
R1(f)	$H_RL_2+L \rightarrow H_RL_3$	kf_{RL}	H_RL_3	Association of H_R L_2 and L creates H_RL_3.
R1(f)	$H_RL_2+L \rightarrow H_RL_3$	kf_{RL}		2nd match for association rule.
R1(b)	$H_RL_3 \rightarrow H_RL_2+L$	kb_{RL}	–	Dissociation of H_R and L.
R1(b)	$H_RL_3 \rightarrow H_RL_2+L$	kb_{RL}	–	2nd match for dissociation rule.
R1(b)	$H_RL_3 \rightarrow H_RL_2+L$	kb_{RL}	–	3rd match for dissociation rule.
R2(f)	$H_TL_2+L \rightarrow H_TL_3$	kf_{TL}	H_TL_3	Association of H_T L_2 and L creates H_TL_3.
R2(f)	$H_TL_2+L \rightarrow H_TL_3$	kf_{TL}		2nd match for association rule.
R2(b)	$H_TL_3 \rightarrow H_TL_2+L$	kb_{TL}	–	Dissociation of H_T and L.
R2(b)	$H_TL_3 \rightarrow H_TL_2+L$	kb_{TL}	–	2nd match for dissociation rule.
R2(b)	$H_TL_3 \rightarrow H_TL_2+L$	kb_{TL}	–	3rd match for dissociation rule.
R1(f)	$H_RL_3+L \rightarrow H_RL_4$	kf_{RL}	H_RL_4	Association of H_R L_3 and L creates H_RL_4.
R1(b)	$H_RL_4 \rightarrow H_RL_3+L$	kb_{RL}	–	Dissociation of H_R and L.
R1(b)	$H_RL_4 \rightarrow H_RL_3+L$	kb_{RL}	–	2nd match for dissociation rule.
R1(b)	$H_RL_4 \rightarrow H_RL_3+L$	kb_{RL}	–	3rd match for dissociation rule.
R1(b)	$H_RL_4 \rightarrow H_RL_3+L$	kb_{RL}	–	4th match for dissociation rule.
R2(f)	$H_TL_3+L \rightarrow H_TL_4$	kf_{TL}	H_TL_4	Association of H_T L_3 and L creates H_TL_4.
R2(b)	$H_TL_4 \rightarrow H_TL_3+L$	kb_{TL}	–	Dissociation of H_T and L.

R2(b)	$H_TL_4 \rightarrow H_TL_3+L$	kb_{TL}	–	2nd match for dissociation rule.
R2(b)	$H_TL_4 \rightarrow H_TL_3+L$	kb_{TL}	–	3rd match for dissociation rule.
R2(b)	$H_TL_4 \rightarrow H_TL_3+L$	kb_{TL}	–	4th match for dissociation rule.
A1(f)	$H_RL \rightarrow H_TL$	$k_{RT}(\Gamma_L)^{\Phi_{LB}}$	–	N=1 and $\Gamma_L = (kf_{TL} / kb_{TL})/(kf_{RL} / kb_{RL})$.
A1(b)	$H_TL \rightarrow H_RL$	$k_{TR}(\Gamma_L)^{\Phi_{LB}-1}$	–	
A1(f)	$H_RL_2 \rightarrow H_TL_2$	$k_{RT}(\Gamma_L)^{2\Phi_{LB}}$	–	N=2.
A1(b)	$H_TL_2 \rightarrow H_RL_2$	$k_{TR}(\Gamma_L)^{2(\Phi_{LB}-1)}$	–	
A1(f)	$H_RL_3 \rightarrow H_TL_3$	$k_{RT}(\Gamma_L)^{3\Phi_{LB}}$	–	N=3.
A1(b)	$H_TL_3 \rightarrow H_RL_3$	$k_{TR}(\Gamma_L)^{3(\Phi_{LB}-1)}$	–	
A1(f)	$H_RL_4 \rightarrow H_TL_4$	$k_{RT}(\Gamma_L)^{4\Phi_{LB}}$	–	N=4.
A1(b)	$H_TL_4 \rightarrow H_RL_4$	$k_{TR}(\Gamma_L)^{4(\Phi_{LB}-1)}$	–	

1.1.3.3 Quartic ternary complex model of a G protein-coupled receptor

This example is the quaternary complex model of GPCR activation which is discussed in the main text of this article (see Figure 5). As shown in Figure 9A and 9B, the model comprises 3 structures and 8 binding rule instances. Table 5 explains how the compilation algorithm applies these rules to create the reaction network shown in Figure 9C.

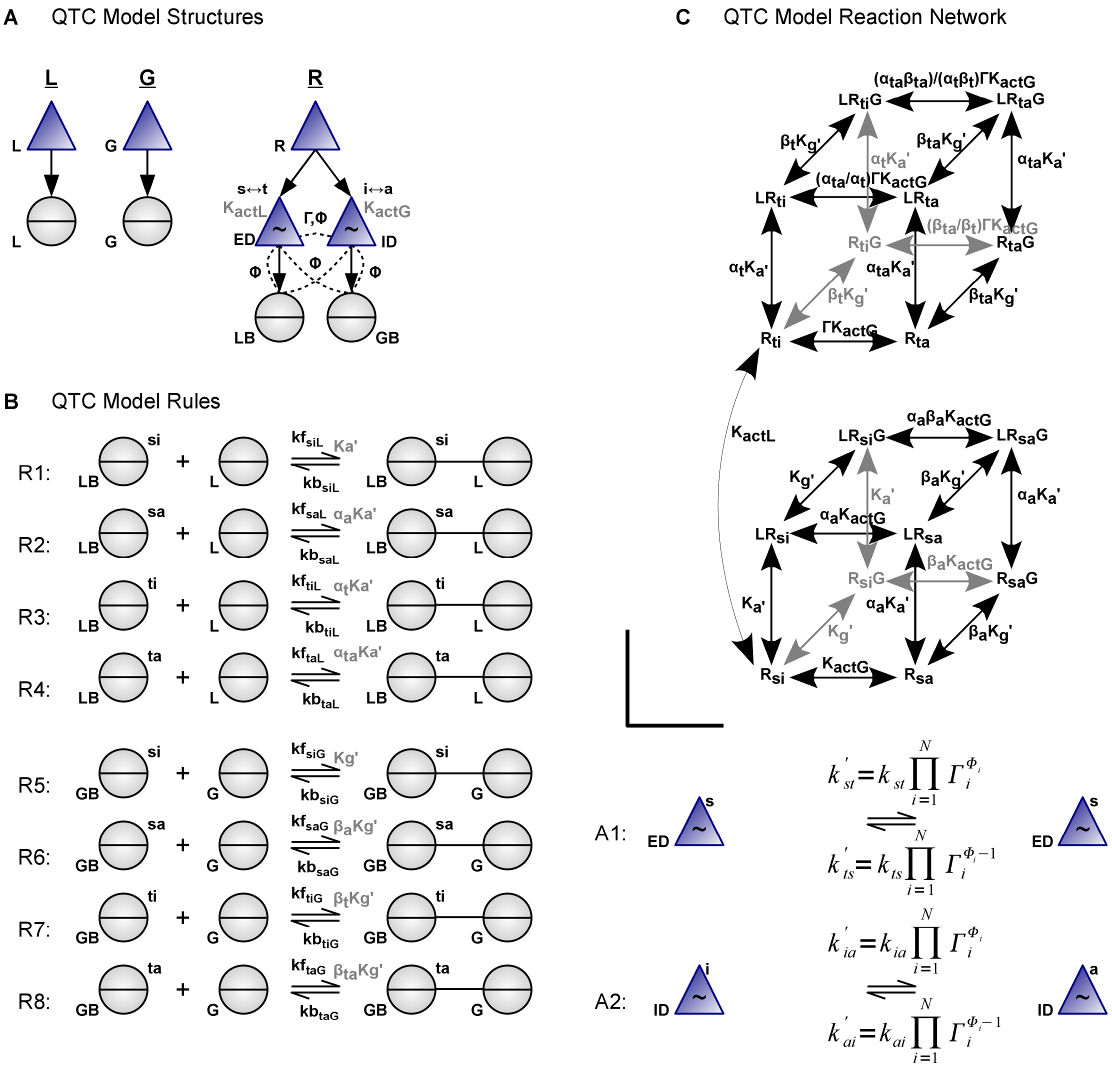

Figure 9: Quartic ternary complex model of a GPCR. A) The model comprises 3 structures named L, G and R, which are instantiated with initial conditions (not shown). The receptor structure R comprises an extracellular, ligand-binding domain ED and an intracellular, G protein-binding domain ID. As shown, the domains are allosterically coupled with regulatory factor Γ and we have annotated allosteric equilibrium constants *KactL* and *KactG*. For simplicity, the effect of all modifiers on both allosteric transitions parameterized by the same Φ-value. **B)** The binding rule instances R1-R8 are explicitly defined in the model. Rules R1-R4 describe the binding of L to each of the 4 conformational states of R. Likewise, R5-R8 describe the binding of G to the 4 conformational states of R. The allosteric transition rule instances A1 and A2 are automatically generated by ANC when the allosteric components ED and ID are defined. To aid comparison with previously published models of GPCR activity and with the reaction network in panel C, we show equilibrium constants (shaded gray) as well as rate constants, with the understanding that the modeller actually supplies rates. **C)** Through the application of the rules, a biochemical reaction network is generated in which the GPCR has 16 possible ligation and conformational states. Edges represent reversible transitions and we have annotated equilibrium constants. For simplicity, only one *s*↔*t* transition is shown.

Table 5: Network generation for the quartic ternary complex model of a GPCR.

This table illustrates how the rule instances in Figure 9 are used by the iterative compilation algorithm to generate binding and allosteric reactions, using the rate constants associated with each rule instance, and creating new structure instances as needed.

Rule applied	Reaction	Rate constant	New species added to network	Notes
Initialization			R_{si}, L, G	We assume the model defines initial conditions for these structure instances (i.e. species).
A1(f)	$R_{si} \rightarrow R_{ti}$	k_{st}	R_{ti}	R is unligated, so there are no modifiers (N=0).
A1(b)	$R_{ti} \rightarrow R_{si}$	k_{ts}	–	R is unligated, so there are no modifiers (N=0).
A2(f)	$R_{si} \rightarrow R_{sa}$	k_{ia}	R_{sa}	R is unligated, so there are no modifiers (N=0).
A2(b)	$R_{sa} \rightarrow R_{si}$	k_{ai}	–	R is unligated, so there are no modifiers (N=0).
A1(f)	$R_{sa} \rightarrow R_{ta}$	$k_{st}(\Gamma)^{\Phi}$	R_{ta}	ID subunit in state *a* modifies ED transition (N=1).
A1(b)	$R_{ta} \rightarrow R_{sa}$	$k_{ts}(\Gamma)^{\Phi-1}$	–	ID subunit in state *a* modifies ED transition (N=1).
A2(f)	$R_{ti} \rightarrow R_{ta}$	$k_{ia}(\Gamma)^{\Phi}$	R_{ta}	ED subunit in state *t* modifies ID transition (N=1).
A2(b)	$R_{ta} \rightarrow R_{ti}$	$k_{ai}(\Gamma)^{\Phi-1}$	–	ED subunit in state *t* modifies ID transition (N=1).
R1(f)	$R_{si}+L \rightarrow LR_{si}$	kf_{siL}	LR_{si}	Association of R_{si} and L creates LR_{si}.
R1(b)	$LR_{si} \rightarrow R_{si}+L$	kb_{siL}	–	Dissociation of R_{si} and L.
R2(f)	$R_{sa}+L \rightarrow LR_{sa}$	kf_{saL}	LR_{sa}	Association of R_{sa} and L creates LR_{sa}.
R2(b)	$LR_{sa} \rightarrow R_{sa}+L$	kb_{saL}	–	Dissociation of R_{sa} and L.
R3f)	$R_{ti}+L \rightarrow LR_{ti}$	kf_{tiL}	LR_{ti}	Association of R_{ti} and L creates LR_{ti}.
R3(b)	$LR_{ti} \rightarrow R_{ti}+L$	kb_{tiL}	–	Dissociation of R_{ti} and L.
R4(f)	$R_{ta}+L \rightarrow LR_{ta}$	kf_{taL}	LR_{ta}	Association of R_{ta} and L creates LR_{ta}.
R4(b)	$LR_{ta} \rightarrow R_{ta}+L$	kb_{taL}	–	Dissociation of R_{ta} and L.
R5(f)	$R_{si}+G \rightarrow R_{si}G$	kf_{siG}	$R_{si}G$	Association of R_{si} and G creates $R_{si}G$.
R5(b)	$R_{si}G \rightarrow R_{si}+G$	kb_{siG}	–	Dissociation of R_{si} and G.
R6(f)	$R_{sa}+G \rightarrow R_{sa}G$	kf_{saG}	$R_{sa}G$	Association of R_{sa} and G creates $R_{sa}G$.
R6(b)	$R_{sa}G \rightarrow R_{sa}+G$	kb_{saG}	–	Dissociation of R_{sa} and G.
R7f)	$R_{ti}+G \rightarrow R_{ti}G$	kf_{tiG}	$R_{ti}G$	Association of R_{ti} and G creates $R_{ti}G$.
R7(b)	$R_{ti}G \rightarrow R_{ti}+G$	kb_{tiG}	–	Dissociation of R_{ti} and G.
R8(f)	$R_{ta}+G \rightarrow R_{ta}G$	kf_{taG}	$R_{ta}G$	Association of R_{ta} and G creates $R_{ta}G$.
R8(b)	$R_{ta}G \rightarrow R_{ta}+G$	kb_{taG}	–	Dissociation of R_{ta} and G.
R1(f)	$R_{si}G+L \rightarrow LR_{si}G$	kf_{siL}	$LR_{si}G$	Association of $R_{si}G$ and L creates $LR_{si}G$.
R1(b)	$LR_{si}G \rightarrow R_{si}G+L$	kb_{siL}	–	Dissociation of $R_{si}G$ and L.
R2(f)	$R_{sa}G+L \rightarrow LR_{sa}G$	kf_{saL}	$LR_{sa}G$	Association of $R_{sa}G$ and L creates $LR_{sa}G$.
R2(b)	$LR_{sa}G \rightarrow R_{sa}G+L$	kb_{saL}	–	Dissociation of $R_{sa}G$ and L.
R3f)	$R_{ti}G+L \rightarrow LR_{ti}G$	kf_{tiL}	$LR_{ti}G$	Association of $R_{ti}G$ and L creates $LR_{ti}G$.
R3(b)	$LR_{ti}G \rightarrow R_{ti}G+L$	kb_{tiL}	–	Dissociation of $R_{ti}G$ and L.
R4(f)	$R_{ta}+L \rightarrow LR_{ta}G$	kf_{taL}	$LR_{ta}G$	Association of $R_{ta}G$ and L creates $LR_{ta}G$.

R4(b)	$LR_{ta}G \rightarrow R_{ta}+L$	kb_{taL}	–	Dissociation of $R_{ta}G$ and L.
R5(f)	$LR_{si}+G \rightarrow LR_{si}G$	kf_{siG}		Association of LR_{si} and G creates $LR_{si}G$.
R5(b)	$LR_{si}G \rightarrow LR_{si}+G$	kb_{siG}	–	Dissociation of LR_{si} and G.
R6(f)	$LR_{sa}+G \rightarrow LR_{sa}G$	kf_{saG}		Association of LR_{sa} and G creates $LR_{sa}G$.
R6(b)	$LR_{sa}G \rightarrow LR_{sa}+G$	kb_{saG}	–	Dissociation of LR_{sa} and G.
R7f)	$LR_{ti}+G \rightarrow LR_{ti}G$	kf_{tiG}		Association of LR_{ti} and G creates $LR_{ti}G$.
R7(b)	$LR_{ti}G \rightarrow LR_{ti}+G$	kb_{tiG}	–	Dissociation of LR_{ti} and G.
R8(f)	$LR_{ta}+G \rightarrow LR_{ta}G$	kf_{taG}		Association of LR_{ta} and G creates $LR_{ta}G$.
R8(b)	$LR_{ta}G \rightarrow LR_{ta}+G$	kb_{taG}	–	Dissociation of LR_{ta} and G.
A1(f)	$LR_{si} \rightarrow LR_{ti}$	$k_{st}(\alpha_t)^{\Phi}$	–	R is ligated to L (N=1).
A1(b)	$LR_{ti} \rightarrow LR_{si}$	$k_{ts}(\alpha_t)^{\Phi-1}$	–	R is ligated to L (N=1).
A2(f)	$LR_{si} \rightarrow LR_{sa}$	$k_{ia}(\alpha_a)^{\Phi}$	–	R is ligated to L (N=1).
A2(b)	$LR_{sa} \rightarrow LR_{si}$	$k_{ai}(\alpha_a)^{\Phi-1}$	–	R is ligated to L (N=1).
A1(f)	$LR_{sa} \rightarrow LR_{ta}$	$k_{st}(\Gamma)^{\Phi}(\alpha_{ta}/\alpha_a)^{\Phi}$	–	R is ligated to L and<br>ID subunit in state *a* modifies ED transition (N=2).
A1(b)	$LR_{ta} \rightarrow LR_{sa}$	$k_{ts}(\Gamma)^{\Phi-1}(\alpha_{ta}/\alpha_a)^{\Phi-1}$	–	R is ligated to L and<br>ID subunit in state *a* modifies ED transition (N=2).
A2(f)	$LR_{ti} \rightarrow LR_{ta}$	$k_{ia}(\Gamma)^{\Phi}(\alpha_{ta}/\alpha_t)^{\Phi}$	–	R is ligated to L and<br>ED subunit in state *t* modifies ID transition (N=2).
A2(b)	$LR_{ta} \rightarrow LR_{ti}$	$k_{ai}(\Gamma)^{\Phi-1}(\alpha_{ta}/\alpha_t)^{\Phi-1}$	–	R is ligated to L and<br>ED subunit in state *t* modifies ID transition (N=2).
A1(f)	$R_{si}G \rightarrow R_{ti}G$	$k_{st}(\beta_t)^{\Phi}$	–	R is ligated to G (N=1).
A1(b)	$R_{ti}G \rightarrow R_{si}G$	$k_{ts}(\beta_t)^{\Phi-1}$	–	R is ligated to G (N=1).
A2(f)	$R_{si}G \rightarrow R_{sa}G$	$k_{ia}(\beta_a)^{\Phi}$	–	R is ligated to G (N=1).
A2(b)	$R_{sa}G \rightarrow R_{si}G$	$k_{ai}(\beta_a)^{\Phi-1}$	–	R is ligated to G (N=1).
A1(f)	$R_{sa}G \rightarrow R_{ta}G$	$k_{st}(\Gamma)^{\Phi}(\beta_{ta}/\beta_a)^{\Phi}$	–	R is ligated to G and<br>ID subunit in state *a* modifies ED transition (N=2).
A1(b)	$R_{ta}G \rightarrow R_{sa}G$	$k_{ts}(\Gamma)^{\Phi-1}(\beta_{ta}/\beta_a)^{\Phi-1}$	–	R is ligated to G and<br>ID subunit in state *a* modifies ED transition (N=2).
A2(f)	$R_{ti}G \rightarrow R_{ta}G$	$k_{ia}(\Gamma)^{\Phi}(\beta_{ta}/\beta_t)^{\Phi}$	–	R is ligated to G and<br>ED subunit in state *t* modifies ID transition (N=2).
A2(b)	$R_{ta}G \rightarrow R_{ti}G$	$k_{ai}(\Gamma)^{\Phi-1}(\beta_{ta}/\beta_t)^{\Phi-1}$	–	R is ligated to G and<br>ED subunit in state *t* modifies ID transition (N=2).
A1(f)	$LR_{si}G \rightarrow LR_{ti}G$	$k_{st}(\alpha_t)^{\Phi}(\beta_t)^{\Phi}$	–	R is ligated to L and G (N=2).
A1(b)	$LR_{ti}G \rightarrow LR_{si}G$	$k_{ts}(\alpha_t)^{\Phi-1}(\beta_t)^{\Phi-1}$	–	R is ligated to L and G (N=2).
A2(f)	$LR_{si}G \rightarrow LR_{sa}G$	$k_{ia}(\alpha_a)^{\Phi}(\beta_a)^{\Phi}$	–	R is ligated to L and G (N=2).
A2(b)	$LR_{sa}G \rightarrow LR_{si}G$	$k_{ai}(\alpha_a)^{\Phi-1}(\beta_a)^{\Phi-1}$	–	R is ligated to L and G (N=2).
A1(f)	$LR_{sa}G \rightarrow LR_{ta}G$	$k_{st}(\Gamma)^{\Phi}(\alpha_{ta}/\alpha_a)^{\Phi}(\beta_{ta}/\beta_a)^{\Phi}$	–	R is ligated to L, G and ID subunit in state *a* modifies ED transition (N=3).

A1(b)	$LR_{ta}G \rightarrow LR_{sa}G$	$k_{ts}(\Gamma)^{\Phi-1}(\alpha_{ta}/\alpha_{a})^{\Phi-1}(\beta_{ta}/\beta_{a})^{\Phi-1}$	–	R is ligated to L, G and ID subunit in state *a* modifies ED transition (N=3).
A2(f)	$LR_{ti}G \rightarrow LR_{ta}G$	$k_{ia}(\Gamma)^{\Phi}(\alpha_{ta}/\alpha_{t})^{\Phi}(\beta_{ta}/\beta_{t})^{\Phi}$	–	R is ligated to L, G and ED subunit in state *t* modifies ID transition (N=3).
A2(b)	$LR_{ta}G \rightarrow LR_{ti}G$	$k_{ai}(\Gamma)^{\Phi-1}(\alpha_{ta}/\alpha_{t})^{\Phi-1}(\beta_{ta}/\beta_{t})^{\Phi-1}$	–	R is ligated to L, G and ED subunit in state *t* modifies ID transition (N=3).

1.2 Derivation of kinetic input-output function

To compute how the kinetics of a component's allosteric transition are affected by the presence of modifiers, we first write the forward and backward kinetic rate constants for the unmodified component in terms of the difference in free energy between the transition state (denoted †) and each conformational state [1]:

$$k_{RT} = Ce^{-\Delta G_{R\dagger}/kT} \tag{1a}$$
$$k_{TR} = Ce^{-\Delta G_{T\dagger}/kT} \tag{1b}$$

where k_{RT} is the kinetic rate for transitioning from the R to the T state and k_{TR} is the kinetic rate for transitioning from the T to the R state. We write the equilibrium constant of the allosteric transition in the unmodified state as

$$K_{RT} = e^{-\Delta G_{RT}/kT} \tag{2}$$

In the presence of N modifiers, we assume that each modifier, indexed by i, contributes independently to the energy of the each conformation and to the energy of the transition state by $\Delta G_R^{(i)}$, $\Delta G_T^{(i)}$ and $\Delta G_\dagger^{(\mathrm{i})}$ respectively, and that the pre-exponential factor C remains constant. Hence, using a prime to indicate the presence of modifiers, we can write:

$$k_{RT}{}' = Ce^{-[\Delta G_{R\dagger}+\sum_{i=1}^{N}(\Delta G_\dagger^{(\mathrm{i})}-\Delta G_\mathrm{R}^{(\mathrm{i})})]/kT} = k_{RT}e^{-\sum_{i=1}^{N}(\Delta G_\dagger^{(\mathrm{i})}-\Delta G_\mathrm{R}^{(\mathrm{i})})/kT} \tag{3a}$$

and similarly

$$k_{TR}{}' = Ce^{-[\Delta G_{T\dagger}+\sum_{i=1}^{N}(\Delta G_\dagger^{(\mathrm{i})}-\Delta G_\mathrm{T}^{(\mathrm{i})})]/kT} = k_{TR}e^{-\sum_{i=1}^{N}(\Delta G_\dagger^{(\mathrm{i})}-\Delta G_\mathrm{T}^{(\mathrm{i})})/kT} \tag{3b}$$

implying that

$$K_{RT}{}' = e^{-[\Delta G_{RT}+\sum_{i=1}^{N}(\Delta G_T^{(i)}-\Delta G_R^{(i)})]/kT} = K_{RT}e^{-\sum_{i=1}^{N}(\Delta G_T^{(i)}-\Delta G_R^{(i)})/kT} = K_{RT}e^{-\sum_{i=1}^{N}\Delta G_{RT}^{(i)}/kT} = K_{RT}\prod_{i=1}^{N}\Gamma_i \tag{4}$$

For convenience, we define a parameter Φ_i as the ratio of the change in the $R \rightarrow T$ activation energy and the change in the free energy of the transition due to the modifier i:

$$\Phi_i = \frac{\Delta G_\dagger^{(\mathrm{i})} - \Delta G_\mathrm{R}^{(\mathrm{i})}}{\Delta G_\mathrm{T}^{(\mathrm{i})} - \Delta G_\mathrm{R}^{(\mathrm{i})}} = \frac{\Delta G_\dagger^{(\mathrm{i})} - \Delta G_\mathrm{R}^{(\mathrm{i})}}{\Delta G_\mathrm{RT}^{(\mathrm{i})}} \tag{5a}$$

and so the corresponding ratio for the T→R transition is

$$\Phi_i - 1 = \frac{\Delta G_\dagger^{(\mathrm{i})} - \Delta G_\mathrm{T}^{(\mathrm{i})}}{\Delta G_\mathrm{RT}^{(\mathrm{i})}} \tag{5b}$$

Hence,

$$k_{RT}{}' = k_{RT}e^{-\sum_{i=1}^{N}(\Delta G_\dagger^{(\mathrm{i})}-\Delta G_\mathrm{R}^{(\mathrm{i})})/kT} = k_{RT}e^{-\sum_{i=1}^{N}\Phi_i\Delta G_\mathrm{RT}^{(\mathrm{i})}/kT} = k_{RT}\prod_{i=1}^{N}(\Gamma_i)^{\Phi_i} \tag{6a}$$

and

$$k_{TR}{}' = k_{TR} e^{-\sum_{i=1}^{N} (\Delta G_{\ddagger}^{(i)} - \Delta G_{T}^{(i)})/kT} = k_{TR} e^{-\sum_{i=1}^{N} (\Phi_i - 1)\Delta G_{RT}^{(i)}/kT} = k_{TR} \prod_{i=1}^{N} (\Gamma_i)^{\Phi_i - 1} \quad (6b)$$

This equation is also equation (8) in the main text.

1.3 The Φ parameter, linear free energy relationships, and independence

Our definition of Φ is consistent with the definition of a free energy relationship

A common assumption used to determine values for rate-constants is to assume that a variation in the free energy of a reaction due to some perturbation generates a proportional variation in the activation energy of the reaction [2]. This assumption implies that there is a linear relationship between the activation energy of a reaction and the free energy change of the reaction.

Our definition of the parameter Φ is consistent with the assumption of a linear free energy relationship to describe the allosteric transition of an unmodified protein and the effects of two modifiers *i* and j on that allosteric transition if and only if $\Phi_i = \Phi_j$

Suppose a linear free energy relationship does exist. We will show that the relationship implies $\Phi_i = \Phi_j$. Let the constants ϕ and a parameterize this relationship. The rate-constant for the transition from the *R* to the *T* conformations of the protein is determined by the activation energy of the transition, and the equilibrium constant for the conformational transition is determined by the free energy of the transition. Thus, we have

$$\log k_{RT} = \phi \log K_{RT} + a \quad ; \quad \log k_{RT}^{\{i\}} = \phi \log K_{RT}^{\{i\}} + a \quad ; \quad \log k_{RT}^{\{j\}} = \phi \log K_{RT}^{\{j\}} + a \quad (10)$$

Here we have defined k_{RT}, $k_{RT}^{\{i\}}$, and $k_{RT}^{\{j\}}$ as the rates describing the transition from the *R* to the *T* state in a protein that has no modifier, only the modifier *i*, and only the modifier *j*, respectively. K_{RT}, $K_{RT}^{\{i\}}$, and $K_{RT}^{\{j\}}$ are the corresponding equilibrium constants. For modifier *i*, the definition of Φ_i, Eq. (5a), can be re-written as

$$\Phi_i = \frac{\log\left(\frac{k_{RT}^{\{i\}}}{k_{RT}}\right)}{\log(\Gamma_i)} \quad (11)$$

using Eqs. (3a) and (4). We can include the free energy associated with the allosteric transition of the protein using the relationship for a thermodynamic cycle, Eq. (6) of the main text, i.e. $K_{RT}^{\{i\}} = K_{RT}\Gamma_i$,

$$\Phi_i = \frac{\log\left(\frac{k_{RT}^{\{i\}}}{k_{RT}}\right)}{\log\left(\frac{K_{RT}^{\{i\}}}{K_{RT}}\right)} \quad (12)$$

or

$$\log k_{RT}^{\{i\}} = \Phi_i \log K_{RT}^{\{i\}} + \log k_{RT} - \Phi_i \log K_{RT} \quad (13)$$

The imposition of a linear free energy relationship, Eqs. (10), and Eq. (13) for both modifiers *i* and *j* implies that

$$\Phi_i = \Phi_j = \phi$$

Alternatively, if we impose $\Phi_i = \Phi_j$ then Eq. (13) implies that a linear free energy relationship exists and is parameterized by $\phi = \Phi_i = \Phi_j$ and $a = \log k_{RT} - \phi \log K_{RT}$.

A linear free energy relationship describing the effect of modifiers applied alone or in combination is equivalent to each modifier contributing independently to the kinetics of the allosteric transition with the same value of Φ

If we assume that a linear free energy relationship describes the effects of two modifiers *i* and *j* on the allosteric transition either alone or in combination, then we expect Eq. (10) to hold and furthermore

$$\log k_{RT}^{\{i,j\}} = \phi \log K_{RT}^{\{i,j\}} + a \tag{15}$$

where $k_{RT}^{\{i,j\}}$ and $K_{RT}^{\{i,j\}}$ are the *R* to *T* rate-constant and the allosteric equilibrium constant of the protein under the combined effect of the two modifiers. Using Eq. (6) of the main text, we can write

$$K_{RT}^{\{i\}} = K_{RT}\Gamma_i \quad ; \quad K_{RT}^{\{j\}} = K_{RT}\Gamma_j \quad ; \quad K_{RT}^{\{i,j\}} = K_{RT}\Gamma_i\Gamma_j \tag{16}$$

Combining Eqs. (10), (15) and (16) gives

$$\log k_{RT}^{\{i\}} = \log k_{RT} + \phi \log \Gamma_i \tag{17a}$$
$$\log k_{RT}^{\{j\}} = \log k_{RT} + \phi \log \Gamma_j \tag{17b}$$
$$\log k_{RT}^{\{i,j\}} = \log k_{RT} + \phi \log \Gamma_i + \phi \log \Gamma_j \tag{17c}$$

and so each modifier contributes independently to the allosteric transition rate, and with the same value of ϕ.

Conversely, if two modifiers contribute independently to the allosteric transition rate, i.e. given

$$\log k_{RT}^{\{i\}} = \log k_{RT} + \Phi_i \log \Gamma_i \tag{18a}$$
$$\log k_{RT}^{\{j\}} = \log k_{RT} + \Phi_j \log \Gamma_j \tag{18b}$$
$$\log k_{RT}^{\{i,j\}} = \log k_{RT} + \Phi_i \log \Gamma_i + \Phi_j \log \Gamma_j \tag{18c}$$

then if we set $\Phi_i = \Phi_j = \phi$ in Eq. (18) and let $a = \log k_{RT} - \phi \log K_{RT}$ we can show using Eq. (16) that

$$\log k_{RT}^{\{i\}} = \phi \log K_{RT}^{\{i\}} + a \tag{19a}$$
$$\log k_{RT}^{\{j\}} = \phi \log K_{RT}^{\{j\}} + a \tag{19b}$$
$$\log k_{RT}^{\{i,j\}} = \phi \log K_{RT}^{\{i,j\}} + a \tag{19c}$$

and so the existence of a linear free energy relationship.

Extension to multiple modifiers is straightforward.

1.4 Validation of ANC using a model of calmodulin

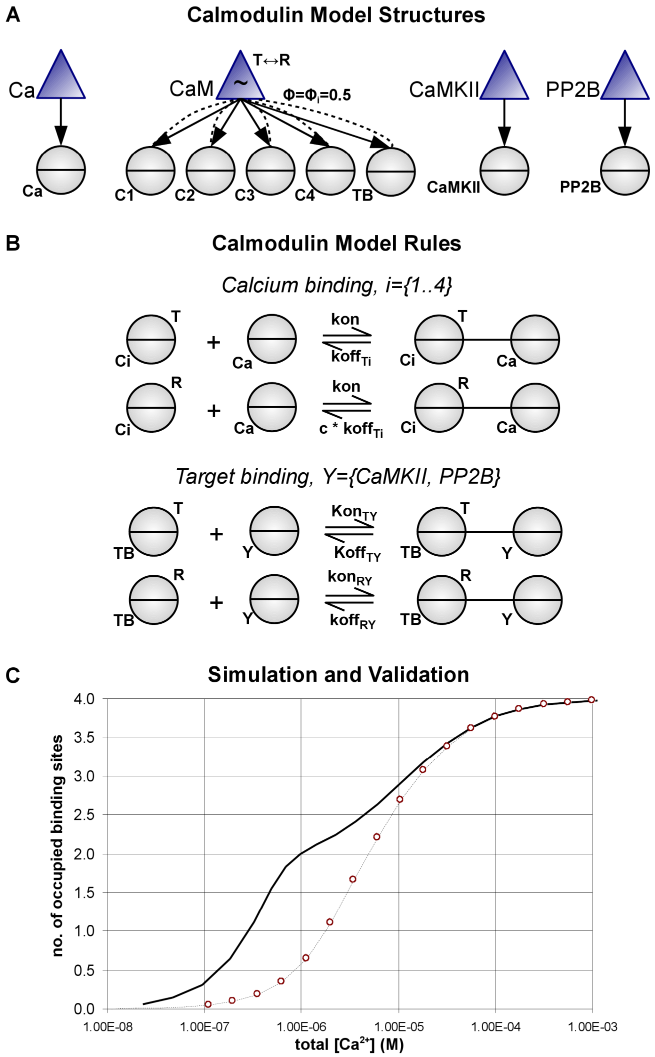

Figure 10: Validation of ANC using a previously published model of calmodulin. (A) ANC-structures of calmodulin, calcium, and the downstream targets of calmodulin – calmodulin-dependent kinase II (CaMKII) or protein phosphatase 2B (PP2P) – implementing the model of Stefan *et al.* [3]. Calmodulin undergoes an allosteric transition between a low affinity state T and a high affinity state R. Calmodulin is a single polypeptide with 4 non-identical calcium-binding sites and a 5th binding site for downstream targets. All ligands modify of the allosteric transition with the same value of Φ. **(B)** Binding rules give the affinity of calcium, CaMKII, and PP2P to their binding sites on calmodulin. Rather than list 6 sets of rules *ad nauseam*, we indicate through the variables *i* and *Y* that the binding rates depend on the binding site and target enzyme involved. The affinity of both downstream targets to the T state is zero in the model of Stefan *et al.* **(C)** ANC correctly generates the 352 uni-directional biochemical reactions given by Stefan *et al*. After exporting the model into *Matlab* using *Facile*, we simulated the system and measured the steady-state Ca^{2+} occupancy (number of bound sites) of calmodulin for various concentrations of Ca^{2+} both in the presence (thick line) and absence (thin line) of 75 μM of CaMKII. We use 0.2 μM of calmodulin and the parameter values of Stefan *et al.* Our simulation results were consistent with the simulations shown in Fig. 3 and 4 of Stefan *et al*. Finally, our simulations also match the theoretical occupancy of calmodulin in the absence of CaMKII (red circles) which we computed using the equation given by Stefan *et al.* (c.f. Equation 1).

2 Supplementary Results

2.1 Mathematical analysis of a generic divalent allosteric protein and two ligands

Here, we analyze the reaction network of Figure 1C of the main article to determine the cooperativity of binding of ligands X and Y and also the apparent affinity of the ligands to A. To do so, we must coarse-grain the network by summing over the conformational states of A, thus obtaining the 4-state diagram of Figure 2A (inset) of the main article, where we have defined the following coarse-grained variables: $A=A_R+A_T$, $XA=XA_R+XA_T$, $AY=A_RY+A_TY$, and $XAY=XA_RY+XA_TY$. We need to calculate the parameters of the coarse-grained model, starting with K_X and referring to Figures 1 and 2 of the main article:

$$K_X = \frac{XA}{A} = \frac{XA_R + XA_T}{A_R + A_T} = \frac{K_{RX} + K_{RX}K_{RT}\Gamma_X}{1+K_{RT}} = K_{RX}\frac{1+K_{RT}\Gamma_X}{1+K_{RT}} \tag{20}$$

Likewise,

$$K_Y = \frac{AY}{A} = \frac{A_RY + A_TY}{A_R + A_T} = \frac{K_{RY} + K_{RY}K_{RT}\Gamma_Y}{1+K_{RT}} = K_{RY}\frac{1+K_{RT}\Gamma_Y}{1+K_{RT}} \tag{21}$$

For the cooperativity parameter θ:

$$\begin{gathered}\theta = \left(\frac{1}{K_Y}\right)\frac{XAY}{XA} = \left(\frac{1}{K_Y}\right)\frac{XA_RY + XA_TY}{XA_R + XA_T} = \\ \left(K_{RY}\frac{1+K_{RT}\Gamma_Y}{1+K_{RT}}\right)^{-1}\frac{K_{RX}K_{RY} + K_{RX}K_{RY}K_{RT}\Gamma_X\Gamma_Y}{K_{RX} + K_{RX}K_{RT}\Gamma_X} = \frac{(1+K_{RT})(1+K_{RT}\Gamma_X\Gamma_Y)}{(1+K_{RT}\Gamma_X)(1+K_{RT}\Gamma_Y)}\end{gathered} \tag{22}$$

Setting $\frac{d\theta}{dK_{RT}} = 0$ allows us to solve for the value of K_{RT} that gives the maximum θ, given by

$$K_{RT}^{\max} = \frac{1}{\sqrt{\Gamma_X\Gamma_Y}} \tag{23}$$

and this yields

$$\theta_{\max} = \frac{\left(1+\frac{1}{\sqrt{\Gamma_X\Gamma_Y}}\right)\left(1+\sqrt{\Gamma_X\Gamma_Y}\right)}{\left(1+\frac{\Gamma_X}{\Gamma_Y}\right)\left(1+\frac{\Gamma_Y}{\Gamma_X}\right)}. \tag{24}$$

2.2 Effect of allosteric cooperativity on the width and maximum response of XAY trimer assembly

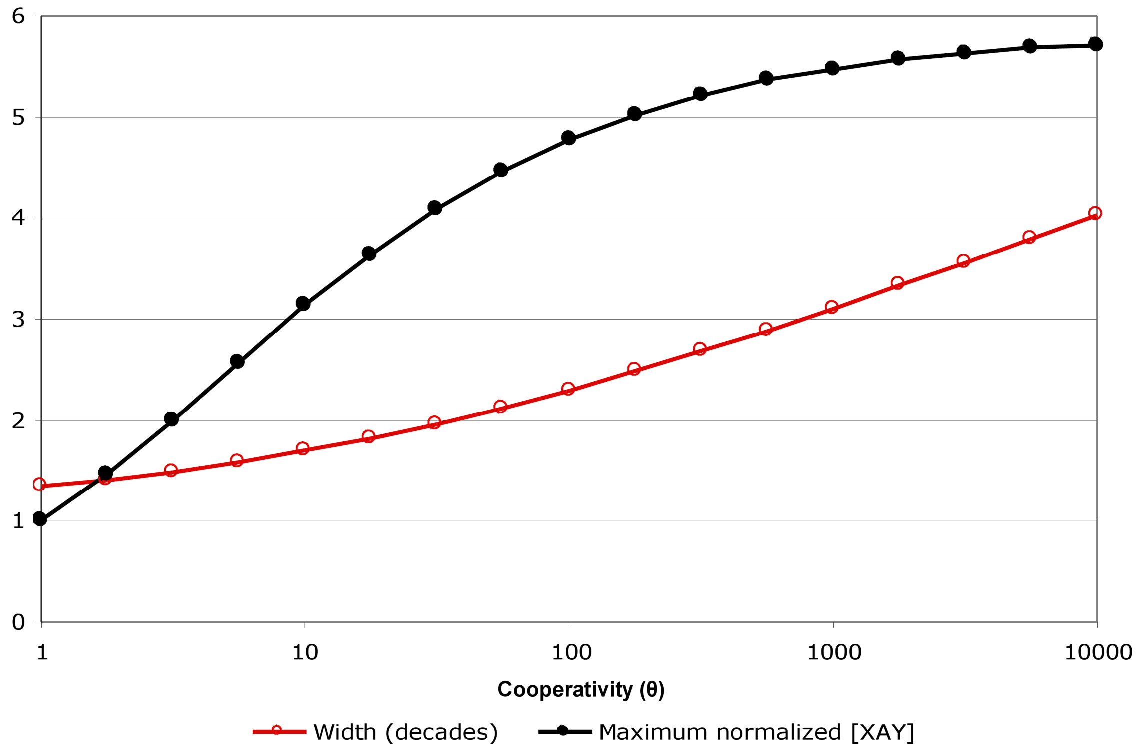

Figure 11: Effect of allosteric cooperativity on the width and maximum response of XAY timer assembly. This plot shows the width and maximal response of each curve in Figure 2A of the main article (as well for curves for intermediate values of θ not shown there). The width is measured as the logarithmic half-maximal width and given in decades. The maximal response is the maximum value of [XAY] for each curve normalized to maximum for the θ=1 curve.

2.3 Effect of competitive ligands on the EC50 of ligand in the concerted and sequential models

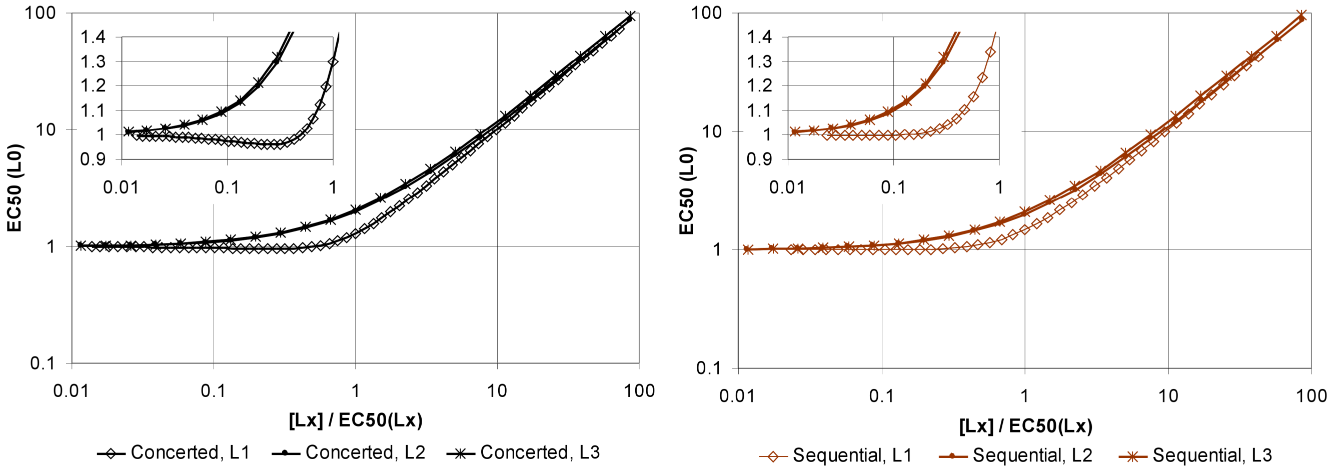

Figure 12: Effect of competitive ligands on the EC50 of ligand in the concerted and sequential models. This plot shows the effect of the competitive ligands L1, L2 and L3 on the EC50 of ligand L0 (see Figure 4 of the main article). The concentration of each competing ligand is normalized to the EC50 of its own occupancy function. The EC50 of L0 is normalized to its EC50 in the absence of a competitor. Only L1 decreases the EC50 of L0, and it does so only slightly and at low concentrations (inset). For the other competitors the EC50 increased monotonically.

2.4 Examples of the allosteric regulation of proteins and receptors by heterogeneous mechanisms

Our modelling framework can describe the allosteric and cooperative interactions ubiquitous in cellular signalling. Ligand binding events, phosphorylation and other post-translational modifications, dimerization and receptor clustering are all examples of mechanisms that can regulate protein interactions. Our framework unifies and simplifies the modelling of such heterogeneous modifiers of protein activity.

For example, dimerization activates the epidermal growth factor receptor (EGFR), a receptor with tyrosine kinase activity [4]. Its extracellular domain binds EGF and other ligands, which induces dimerization of the receptor, followed by autophosphorylation of specific tyrosines on its cytoplasmic domain. As shown in Supplementary Figure 13A and 13B, we can straightforwardly implement in ANC a simplified two-state model of the agonist-induced dimerization of the mutant EGFR characterized by Ozcan *et al.* [5].

Many proteins are also regulated by a combination of ligand-binding and post-translational modifications. For example, the receptors involved in bacterial chemotaxis possess multiple methylation sites whose state of methylation modulates transitions between the bacterium's swimming and tumbling states and so allows adaptation to ambient concentrations of chemoattractants. We have implemented a general model of such receptors in ANC (Figure 13A and 13B). Our approach combines the models of Asakura and Honda [6] and Barkai and Leibler [7], but removes a number of assumptions made to reduce the combinatorial complexity of the system. For instance, we need not assume that methylation and de-methylation occurs in a definite order [6] or that the methylation sites are identical [7]. Concerted allosteric models of clusters of chemotaxis receptors have also been proposed [8] and can be straightforwardly implemented in ANC.

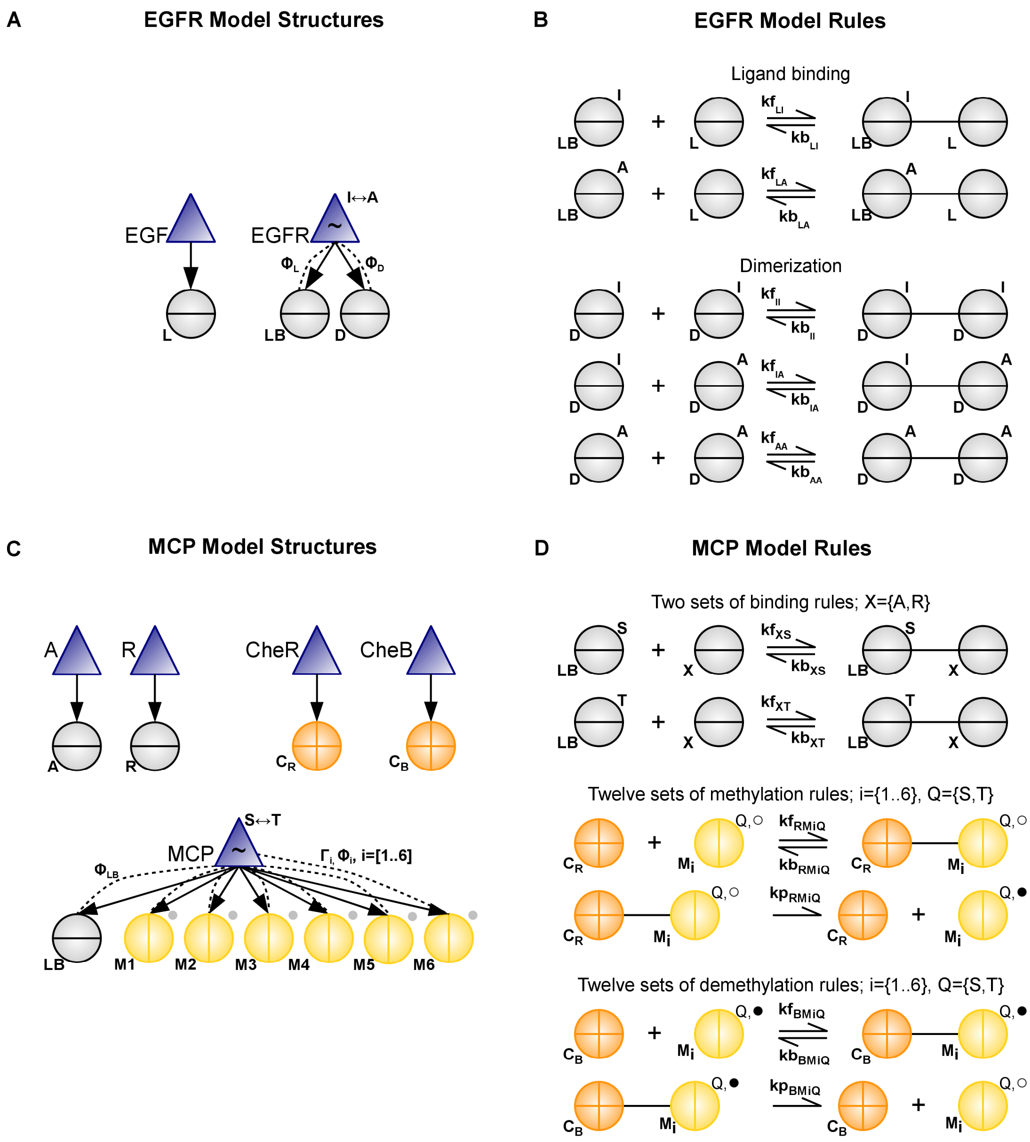

Figure 13: Modelling the regulation of receptors by heterogeneous inputs. (A) Epidermal Growth Factor Receptor (EGFR). The receptor transitions between an inactive (*I*) state and an active (*A*) state and the transition rate is modified by EGF binding or dimerization and with the indicated Φ-values. Its ligand has a single receptor-binding component. **(B)** Two rules specify the rates of ligand binding with each state of the receptor and three rules give dimerization rates for each combination of receptor conformations (*II*, *IA*, and *AA*). **(C)** The methyl-accepting chemotaxis (MCP) proteins are receptors with one ligand-binding (LB) site for either an attractant or a repellent and 6 methyl-accepting sites. Gray circles are placeholders for the methylation state. Both the ligand-binding site and the methylation sites are modifiers of the allosteric equilibrium between the swim (*S*) and tumble (*T*) conformations of the receptor. The attractant A binds and favours the *S* form of the MCP while the repellent R binds and favours the *T* state. Two enzymes, CheR and CheB, methylate and de-methylate the modification sites. Increasing methylation of the MCP favours the *T* state. **(D)** Rules for the binding of A and R to the LB site and enzymatic rules for methylation and de-methylation (c.f. Figure 1B of the main article). Rather than list 24 almost-identical rules *ad nauseam*, we indicate through the variables *i* and *Q* that the rates of (de)methylation depend both on the the methylation site (given by *i*), and the conformational state of the receptor (given by *Q*). Naturally, in the textual form of the model each of the 24 rules is given explicitly.

2.5 Regulatory complexity

In ANC, regulatory interactions are best modelled using allosteric components, which embody Monod *et al.*'s paradigm of allostery: an allosteric component exists in 2 interconvertible conformational states, and modifiers interact non-cooperatively with each state but bias the equilibrium between conformations [9]. Alternatively, with ANC or other rule-based tools, regulation can also be modelled in an *ad hoc* fashion where we explicitly encode regulatory logic in rules (e.g. a rule that says to bind Y only if X is bound).

What are the advantages of each method in terms of model complexity? *Ad hoc* rules appear simpler initially because they require less species. As the size of the system grows, however, such rules may generate regulatory complexity. In an *ad hoc*, interaction-centric approach, we generally cannot make any *a priori* assumptions to simplify a model, such as a particular mechanism for allostery or that some ligated states are not significantly populated. We must therefore specify affinity and cooperativity parameters for a combinatorial number of ligated states. In ANC, however, we assume that the protein has only two conformations and that a ligand's affinity depends only on the conformation, and not on the state of ligation of the protein.

2.5.1 Equilibrium analysis

Consider a protein having N binding sites, with each binding site having L_k (k=0..N-1) distinct ligands, and with all states of ligation and transitions between ligated states possible.

2.5.1.1 Ad hoc approach

Number of independent parameters

To compute the number of independent parameters *(P)* involved in building a model of cooperative ligand binding for this protein, we first recognize that the reaction network induced by ligand binding has thermodynamic cycles in which any equilibrium constant in the cycle can be calculated if the others are known. We proceed to construct the reaction network starting from the unligated protein. First consider the binding of a single ligand. Each ligand will have a distinct affinity to the unligated protein. Next, each pair of ligands that bind generates a 4-sided thermodynamic cycle in which the affinity of each ligand binding to the unligated state is known. In this cycle, an independent cooperativity parameter describes how each ligand affects the other's binding and allows the calculation of the 2 unknown affinities. For each distinct triplet of ligands, we need only consider three 4-sided cycles to generate all states leading to the formation of the tri-liganded protein. The reaction network for these states lies on adjacent sides of a cube. We again assign a cooperativity parameter to each cycle. However, since these three cycles share sides, only one cooperativity parameter is unique. Thus, knowing the reaction network for any two ligands, we can compute the equilibrium constants for the reaction network for three ligands with only a single additional cooperativity parameter per triplet of ligands. Similarly, we can compute the reaction network for up to (k+1) ligands given only a single cooperativity parameter per (k+1)-tuplet of ligands if we already know the reaction network for k ligands. Thus, by induction we can compute *P* by counting the number of ligand combinations for each binding site, each pair of binding sites, each triplet of binding sites etc. Hence:

$$P = \sum_{i_0=0}^{N-1} L_{i_0} + \sum_{i_0=0}^{N-2} \sum_{i_1=i_0+1}^{N-1} L_{i_0} L_{i_1} + \sum_{i_0=0}^{N-3} \sum_{i_1=i_0+1}^{N-2} \sum_{i_2=i_1+1}^{N-1} L_{i_0} L_{i_1} L_{i_2} + \ldots = \prod_{k=0}^{N-1} (1+L_k) - 1 \qquad (30)$$

In the case where L_k=L, *P* reduces to:

$$\mathrm{P} = (1+\mathrm{L})^{\mathrm{N}} - 1 \qquad (31)$$

Number of dependent parameters

The number of dependent parameters may impact on the performance of a rule-based algorithm which has to calculate them, and this number will also help determine how many biochemical equations are in the reaction network described by a model.

Each unique n-tuple of bound ligands has n associated affinity constants giving the affinity of each ligand to the corresponding (n-1)-tuple. Thus, the total number of affinity parameters is:

$$P_T = (1)\sum_{i_0=0}^{N-1} L_{i_0} + (2)\sum_{i_0=0}^{N-2}\sum_{i_1=i_0+1}^{N-1} L_{i_0}L_{i_1} + (3)\sum_{i_0=0}^{N-3}\sum_{i_1=i_0+1}^{N-2}\sum_{i_2=i_1+1}^{N-1} L_{i_0}L_{i_1}L_{i_2} + \dots \quad (32)$$

and the number of dependent parameters is:

$$p = P_T - P = (1)\sum_{i_0=0}^{N-2}\sum_{i_1=i_0+1}^{N-1} L_{i_0}L_{i_1} + (2)\sum_{i_0=0}^{N-3}\sum_{i_1=i_0+1}^{N-2}\sum_{i_2=i_1+1}^{N-1} L_{i_0}L_{i_1}L_{i_2} + \dots \quad (33)$$

For the case wherein L_k=L, we note that since each term of the above summation terms corresponds to singlets, pairs, triplets etc. of binding sites, then the k_{th} such summation term has $\binom{N}{k}$ individual terms. Therefore:

$$P_T = (1)\binom{N}{1}L + (2)\binom{N}{2}L^2 + \dots + (N-1)\binom{N}{N-1}L^{N-1} + (N)\binom{N}{N}L^N \quad (34)$$

To evaluate this expression, we differentiate the binomial theorem with respect to L and multiply both sides by L:

$$(1+L)^N = \sum_{k=0}^{N}\binom{N}{k}L^k \quad (35)$$

$$LN(1+L)^{N-1} = \sum_{k=1}^{N} k\binom{N}{k}L^k \quad (36)$$

Then comparison with P_T yields:

$$\mathrm{P_T} = \mathrm{LN}(1+\mathrm{L})^{\mathrm{N-1}} \quad (37)$$

We can now compute p:

$$\mathrm{p} = \mathrm{P_T} - \mathrm{P} = \mathrm{LN}(1+\mathrm{L})^{\mathrm{N-1}} - (1+\mathrm{L})^{\mathrm{N}} + 1 = (LN - 1 - L)(1+\mathrm{L})^{\mathrm{N-1}} + 1 \quad (38)$$

Number of species

We have L_k+1 occupancy choices at each of N binding sites, and so the number of species *S* is

$$S = \prod_{k=0}^{N-1}(L_k + 1) \quad (39)$$

In the case where L_k=L, we have that S = (L + 1)^N.

2.5.1.2 ANC approach

Following Monod et al., we assume that the protein undergoes concerted transitions between two conformational states and that the affinity of ligands depends only on the conformer they bind. Therefore, the number of independent parameters consists of 2 affinity parameters per ligand, plus an allosteric equilibrium parameter:

Number of independent parameters

$$P = 2\sum_{k=0}^{N-1} L_k + 1 \qquad (40)$$

If the number of ligands is the same for each site (L_k=L), then *P* reduces to

$$P = 2NL + 1 \qquad (41)$$

Number of dependent parameters

The only dependent parameters are the allosteric equilibrium parameters which are associated with each ligated state of the protein. They are dependent because they can be calculated by considering the thermodynamic cycles induced by ligand binding. Indeed, the calculation for a given ligation state is to multiply the baseline (unligated) allosteric equilibrium constant with the ratio of the affinities of each ligand to the 2 conformers of the protein. Hence the number of dependent equilibrium parameters is equal to the number of ligated states:

$$p = \sum_{i_0=0}^{N-1} L_{i_0} + \sum_{i_0=0}^{N-2} \sum_{i_1=i_0+1}^{N-1} L_{i_0} L_{i_1} + \sum_{i_0=0}^{N-3} \sum_{i_1=i_0+1}^{N-2} \sum_{i_2=i_1+1}^{N-1} L_{i_0} L_{i_1} L_{i_2} + \ldots = \prod_{k=0}^{N-1} (1 + L_k) - 1 \qquad (42)$$

As before, in the case that L_k=L,

$$p = (1 + L)^N - 1 \qquad (43)$$

Number of species

Since there are two conformers per occupancy state, the number of species is double that obtained for the *ad hoc* case.

$$S = 2\prod_{k=0}^{N-1} (L_k + 1) \qquad (44)$$

In the case where L_k=L, we have that $S = 2(L + 1)^N$.

2.5.2 *Including kinetic rates*

For this analysis, we assume that all the relevant equilibrium affinities are known.

2.5.2.1 Ad hoc approach

One independent kinetic rate must be specified for each equilibrium constant. Therefore the number of independent kinetic parameters Q is

$$Q = P_T = (1)\sum_{i_0=0}^{N-1} L_{i_0} + (2)\sum_{i_0=0}^{N-2}\sum_{i_1=i_0+1}^{N-1} L_{i_0}L_{i_1} + (3)\sum_{i_0=0}^{N-3}\sum_{i_1=i_0+1}^{N-2}\sum_{i_2=i_1+1}^{N-1} L_{i_0}L_{i_1}L_{i_2} + \dots \quad (50)$$

If $L_k = L$ then

$$Q = LN(1 + L)^{N-1} \quad (51)$$

2.5.2.2 ANC approach

We assume that not only the affinity but also the association and dissociation kinetic rate constants associated with each ligand depend only on the protein's conformation. We also need one kinetic parameter for each allosteric transition. Hence, the number of independent kinetic parameters required is (assuming equilibrium parameters are known):

$$Q = P + p = 2\sum_{k=0}^{N-1} L_k + \prod_{k=0}^{N-1}(1 + L_k) \quad (52)$$

If $L_k = L$, this is:

$$Q = 2NL + (1 + L)^N \quad (53)$$

If we further assume that each ligand contributes independently to the kinetics of the allosteric transition, which we can then compute for any ligated state given a Φ parameter associated with each ligand. Then, the expression for Q becomes:

$$Q = P + \sum_{k=0}^{N-1} L_k = 3\sum_{k=0}^{N-1} L_k + 1 \quad (54)$$

Again, if $L_k = L$, this is:

$$Q = 3NL + 1 \quad (55)$$

We note that this assumption of independence allowed us to drop from a combinatorial number of kinetic parameters to a linear one.

Finally, a standard assumption of the effect of orthosteric ligands on allosteric kinetics is that a linear free energy relation exists between the free energy change in the allosteric equilibrium and the free energy of the transition state. This relationship implies that only a single Φ parameter needs to be specified for orthosteric ligands. Under this assumption, the number of independent rate constants drops to:

$$Q = P + N = 2\sum_{k=0}^{N-1} L_k + 1 + N \quad (56)$$

With $L_k = L$, we obtain:

$$Q = 2NL + 1 + N \tag{57}$$

2.5.3 Discussion of regulatory complexity

We have analyzed the regulatory complexity of a protein having N binding sites indexed by *k* and L_k ligands binding at each site. We did so for the interaction-centric, *ad hoc* approach, in which we made no assumptions concerning the underlying mechanism for cooperative binding of ligands, and for ANC's approach, where we assumed a two-state model and that ligands interact independently with a protein's conformational states. Table 1 summarizes the results of our analysis, giving both the combinatorial and regulatory complexity for the cases we analyzed. We see that the number of independent equilibrium and kinetic parameters scales combinatorially in the *ad hoc* approach, but only linearly in ANC. Nevertheless, the number of states of the protein, or its combinatorial complexity, doubles in ANC. In Table 2, we put in numbers to show that the *ad hoc* approach is advantageous only for monovalent proteins or divalent proteins with at most two ligands.

2.5.4 *Summary of regulatory complexity analysis*

Table 6 - Summary of Equations from Regulatory Complexity Analysis

**Case**	**No. Species and Equilibrium Params (Ad Hoc)**	**No. Species and Equilibrium Params (ANC)**	**No. Kinetic Params (Ad hoc)**	**No. Kinetic Params (ANC)**
General case:<br><br>N-valent<br>Site k has L_k ligands	$S=\prod_{k=0}^{N-1}(L_k+1)$<br>$P=\prod_{k=0}^{N-1}(1+L_k)-1$<br>$p=(1)\sum_{i_0=0}^{N-2}\sum_{i_1=i_0+1}^{N-1}L_{i_0}L_{i_1}+$<br>$(2)\sum_{i_0=0}^{N-3}\sum_{i_1=i_0+1}^{N-2}\sum_{i_2=i_1+1}^{N-1}L_{i_0}L_{i_1}L_{i_2}+\ldots$	$S=2\prod_{k=0}^{N-1}(L_k+1)$<br>$P=2\sum_{k=0}^{N-1}L_k+1$<br>$p=\prod_{k=0}^{N-1}(1+L_k)-1$	$Q=(1)\sum_{i_0=0}^{N-1}L_{i_0}+(2)\sum_{i_0=0}^{N-2}\sum_{i_1=i_0+1}^{N-1}L_{i_0}L_{i_1}+$<br>$(3)\sum_{i_0=0}^{N-3}\sum_{i_1=i_0+1}^{N-2}\sum_{i_2=i_1+1}^{N-1}L_{i_0}L_{i_1}L_{i_2}+\ldots$	$Q=3\sum_{k=0}^{N-1}L_k+1$<br><br>With LFER assumption:<br><br>$Q=2\sum_{k=0}^{N-1}L_k+1+N$
General case with L_k=L	$S = (L + 1)^N$<br>$P=(1+L)^N-1$<br>$p=(LN-1-L)(1+L)^{N-1}+1$	$S = 2(L + 1)^N$<br>$P=2NL+1$<br>$p=(1+L)^N-1$	$Q = LN(1 + L)^{N-1}$	Q = 3NL + 1<br><br>With LFER assumption:<br><br>Q = 2NL + 1 + N
Below, we evaluate the general formulas for two useful special cases				
Special case #1:<br><br>N-valent, L=1	$S = 2^N$<br>$P = 2^N - 1$<br>$p = (N - 2) * 2^{N-1} + 1$	$S = 2^{N+1}$<br>P = 2N + 1<br>$p = 2^N - 1$	$Q = N2^{N-1}$	Q = 3N + 1
Special case #2:<br><br>Bi-valent (N=2)	$P = L_0 + L_1 + L_0L_1$<br>$p = L_0L_1$<br>$S = (L_0 + 1)(L_1 + 1)$<br><br>If L_k=L:<br><br>$P = 2L + L^2$<br>$p = L^2$<br>$S = (L + 1)^2$	$P = 2(L_0 + L_1) + 1$<br>$p = L_0 + L_1 + L_0L_1$<br>$S = 2(L_0 + 1)(L_1 + 1)$<br><br>If L_k=L:<br><br>P = 4L + 1<br>$p = (1 + L)^2 - 1$<br>$S = 2(L + 1)^2$	$Q = 2(L^2 + L)$	Q = 6L + 1<br><br>With LFER assumption:<br><br>Q = 4L + 3

2.5.5 *Comparison of interaction-centric and biomolecule-centric approaches*

Table 7: Comparison of Interaction-Centric and Biomolecule-Centric Approaches

This table uses the formulas in Table 1 for the general case with L_k=L to illustrate regulatory complexity of an N-valent protein given various values of L and N. The *ad hoc* approach is more parsimonious in parameters only for divalent proteins with at most 2 orthosteric ligands per site (orange boxes).

Number of equilibrium parameters required by *ah hoc* approach

	L				
**N**	**1**	**2**	**3**	**4**	**5**
**1**	1	2	3	4	5
**2**	3	8	15	24	35
**3**	7	26	63	124	215
**4**	15	80	255	624	1295
**5**	31	242	1023	3124	7775
**6**	63	728	4095	15624	46655

Number of kinetic parameters required by *ah hoc* approach

	L				
**N**	**1**	**2**	**3**	**4**	**5**
**1**	1	2	3	4	5
**2**	4	12	24	40	60
**3**	12	54	144	300	540
**4**	32	216	768	2000	4320
**5**	80	810	3840	12500	32400
**6**	192	2916	18432	75000	233280

Number of equilibrium parameters required by ANC approach

	L				
**N**	**1**	**2**	**3**	**4**	**5**
**1**	3	5	7	9	11
**2**	5	9	13	17	21
**3**	7	13	19	25	31
**4**	9	17	25	33	41
**5**	11	21	31	41	51
**6**	13	25	37	49	61

Number of kinetic parameters required by ANC approach

	L				
**N**	**1**	**2**	**3**	**4**	**5**
**1**	4	7	10	13	16
**2**	7	13	19	25	31
**3**	10	19	28	37	46
**4**	13	25	37	49	61
**5**	16	31	46	61	76
**6**	19	37	55	73	91

Number of excess equilibrium parameters of *ad hoc* approach over ANC approach

	L				
**N**	**1**	**2**	**3**	**4**	**5**
**1**	-2	-3	-4	-5	-6
**2**	-2	-1	2	7	14
**3**	0	13	44	99	184
**4**	6	63	230	591	1254
**5**	20	221	992	3083	7724
**6**	50	703	4058	15575	46594

Number of excess kinetic parameters of *ad hoc* approach over ANC approach

	L				
**N**	**1**	**2**	**3**	**4**	**5**
**1**	-3	-5	-7	-9	-11
**2**	-3	-1	5	15	29
**3**	2	35	116	263	494
**4**	19	191	731	1951	4259
**5**	64	779	3794	12439	32324
**6**	173	2879	18377	74927	233189

2.6 Derivation of QTC to CTC mapping functions

Here we derive the mapping functions for the projection of our quartic ternary complex model onto Weiss et al.'s cubic ternary complex model. The states of the cubic model are: (R_i, R_a), while the quartic model has (R_{si}, R_{ti}, R_{sa}, R_{ta}). Each model's parameters are given in Figure 5 in the main article. We define a mapping where $R_i=R_{si}+R_{ti}$ and $R_a=R_{sa}+R_{ta}$. Given this mapping, we wish to express the parameters of the cubic model in terms of those of the quartic model. We start with the allosteric equilibrium parameter K_{act}, which is the ratio of unligated R_a and R_i:

$$K_{act} \equiv \frac{R_a}{R_i} = \frac{R_{sa}+R_{ta}}{R_{si}+R_{ti}} = \frac{GR_{si}+\Gamma LGR_{si}}{R_{si}+LR_{si}} = G\frac{1+\Gamma L}{1+L} \tag{60}$$

Next, K_a is the ratio of ligated and unligated R_i (the superscript L indicates a ligated state):

$$K_a \equiv \frac{R_i^L}{R_i} = \frac{R_{si}^L+R_{ti}^L}{R_{si}+R_{ti}} = \frac{K_a^{'}R_{si}+\alpha_t K_a^{'}LR_{si}}{(1+L)R_{si}} = K_a^{'}\frac{1+\alpha_t L}{1+L} \tag{61}$$

Similarly, K_g is

$$K_g \equiv \frac{R_i^G}{R_i} = \frac{R_{si}^G+R_{ti}^G}{R_{si}+R_{ti}} = \frac{K_g^{'}R_{si}+\beta_t K_g^{'}LR_{si}}{(1+L)R_{si}} = K_g^{'}\frac{1+\beta_t L}{1+L} \tag{62}$$

The differential affinity, α, of L for each state:

$$\alpha \equiv \frac{R_a^L/R_a}{R_i^L/R_i} = \frac{1}{Kact}\frac{R_a^L}{R_i^L} = \frac{1}{Kact}\frac{R_{sa}^L+R_{ta}^L}{R_{si}^L+R_{ti}^L} = \frac{1}{Kact}\frac{\alpha_a K_a^{'}GR_{si}+\alpha_{ta}K_a^{'}LG\Gamma R_{si}}{K_a^{'}R_{si}+\alpha_t K_a^{'}LR_{si}}$$
$$= \left(\frac{1+L}{1+\Gamma L}\right)\frac{\alpha_a+\alpha_{ta}\Gamma L}{1+\alpha_t L} \tag{63}$$

And similarly, the differential affinity β:

$$\beta \equiv \frac{R_a^G/R_a}{R_i^G/R_i} = \frac{1}{Kact}\frac{R_a^G}{R_i^G} = \frac{1}{Kact}\frac{R_{sa}^G+R_{ta}^G}{R_{si}^G+R_{ti}^G} = \frac{1}{Kact}\frac{\beta_a K_g^{'}GR_{si}+\beta_{ta}K_g^{'}LG\Gamma R_{si}}{K_g^{'}R_{si}+\beta_t K_g^{'}LR_{si}} = \left(\frac{1+L}{1+\Gamma L}\right)\frac{\beta_a+\beta_{ta}\Gamma L}{1+\beta_t L} \tag{64}$$

For the binding cooperativity parameter, γ, the affinity of G to R_i^L is (γ K_g), hence:

$$\gamma = \frac{1}{K_g}\frac{R_i^{LG}}{R_i^L} = \frac{1}{K_g}\frac{R_{si}^{LG}+R_{ti}^{LG}}{R_{si}^L+R_{ti}^L} = \frac{1}{K_g}\frac{K_a^{'}K_g^{'}R_{si}+\alpha_t K_a^{'}\beta_t K_g^{'}LR_{si}}{K_a^{'}R_{si}+\alpha_t K_a^{'}LR_{si}} = \left(\frac{1+L}{1+\beta_t L}\right)\frac{1+\alpha_t\beta_t L}{1+\alpha_t L} \tag{65}$$

For the activation cooperativity parameter, δ, the equilibrium between R_i^{LG} and R_a^{LG} is given by $\delta\alpha\beta K_{act}$, therefore:

$$\delta = \frac{1}{\alpha\beta K_{act}}\frac{R_a^{LG}}{R_i^{LG}} = \frac{1}{\alpha\beta K_{act}}\frac{R_{sa}^{LG}+R_{ta}^{LG}}{R_{si}^{LG}+R_{ti}^{LG}} = \frac{1}{\alpha\beta K_{act}}\frac{\alpha_a K_a^{'}\beta_a K_g^{'}GR_{si}+\alpha_{ta}K_a^{'}\beta_{ta}K_g^{'}\Gamma LGR_{si}}{K_a^{'}K_g^{'}R_{si}+\alpha_t K_a^{'}\beta_t K_g^{'}LR_{si}}$$
$$= \frac{G}{\alpha\beta K_{act}}\frac{\alpha_a\beta_a+\alpha_{ta}\beta_{ta}\Gamma L}{1+\alpha_t\beta_t L} \tag{66}$$

2.7 ANC Model of Adaptor Protein

Below is the complete model of the adaptor protein of Figure 1C in the main article (and Figure 7 of this document) in a textual form suitable for input to ANC. This model and others mentioned in this work are available online at *http://swainlab.bio.ed.ac.uk/anc*.

```
#############################################################################
# File: adaptor_generic.mod
#
# This example consists of a generic, divalent adapter protein A
# with an input binding site (AX) and an output binding site (AY).
#
# When unliganded, the adapter protein prefers the low-affinity (R) state.
# A modulator X binds to the input site of the adapter more strongly
# in its high-affinity (T) form than in its R form, changing the
# allosteric equilibrium in favour of the active form
#
# Likewise, the target protein Y binds the adapter weakly in
# its low-affinity form, but strongly in its high-affinity form.
#
# Thus, X and Y bind with positive cooperatively to the adaptor.
#
#############################################################################

###################################
MODEL:
###################################

#------------------------------------------------------
# COMPILE PARAMETERS
#------------------------------------------------------
$max_species = -1;

#------------------------------------------------------
# MODEL PARAMETERS
#------------------------------------------------------
# ALLOSTERY
Parameter : {
      name => "kf_RT",
      value => 0.1,
}
Parameter : {
      name => "kb_RT",
      value => 100.0,
}
Parameter : {
      name => "Phi_X",
      value => 0.5,
}
Parameter : {
      name => "Phi_Y",
      value => 0.5,
}

# LIGAND BINDING
Parameter : {
      name => "kf_RX",
```

```
      value => 1.0,
}
Parameter : {
      name => "kb_RX",
      value => 10.0,
}
Parameter : {
      name => "kf_TX",
      value => 10.0,
}
Parameter : {
      name => "kb_TX",
      value => 1.0,
}
Parameter : {
      name => "kf_RY",
      value => 0.01,
}
Parameter : {
      name => "kb_RY",
      value => 1.0,
}
Parameter : {
      name => "kf_TY",
      value => 1.0,
}
Parameter : {
      name => "kb_TY",
      value => 0.01,
}

#------------------------------------------------------
# ADAPTOR PROTEIN
#------------------------------------------------------
ReactionSite: {
      name => "AX",
      type => "bsite",
}
ReactionSite: {
      name => "AY",
      type => "bsite",
}
AllostericStructure: {
      name => A,
      elements => [AX, AY],
      allosteric_transition_rates => [kf_RT, kb_RT],
      allosteric_state_labels => ['R','T'],
      Phi => [Phi_X, Phi_Y],
}

#------------------------------------------------------
# LIGANDS X and Y
#------------------------------------------------------
ReactionSite : {
      name => "X",
      type => "bsite",
}
```

```
Structure: {name => X, elements => [X]}

ReactionSite : {
      name => "Y",
      type => "bsite",
}
Structure: {name => Y, elements => [Y]}

#------------------------------------------------------
# RULES
#------------------------------------------------------
CanBindRule : {
      ligand_names => ['X', 'AX'],
      ligand_allosteric_labels => ['.', 'R'],
      kf => kf_RX,
      kb => kb_RX,
}

CanBindRule : {
      ligand_names => ['X', 'AX'],
      ligand_allosteric_labels => ['.', 'T'],
      kf => kf_TX,
      kb => kb_TX,
}

CanBindRule : {
      ligand_names => ['Y', 'AY'],
      ligand_allosteric_labels => ['.', 'R'],
      kf => kf_RY,
      kb => kb_RY,
}

CanBindRule : {
      ligand_names => ['Y', 'AY'],
      ligand_allosteric_labels => ['.', 'T'],
      kf => kf_TY,
      kb => kb_TY,
}

#------------------------------------------------------
# PROBES
#------------------------------------------------------

Probe : {
      name => "TRIMER",
      classes => ComplexInstance,
      filters => [
            '$_->get_num_elements() == 3',
        ],
}

Probe : {
      name => "AX_DIMER",
      classes => ComplexInstance,
      filters => [
            '$_->get_num_elements() == 2',
            '$_->get_exported_name() =~ /A.*X/',
```

```
        ],
}

Probe : {
      name => "AY_DIMER",
      classes => ComplexInstance,
      filters => [
            '$_->get_num_elements() == 2',
            '$_->get_exported_name() =~ /A.*Y/',
        ],
}

Probe : {
      name => "A",
      classes => ComplexInstance,
      filters => [
            '$_->get_num_elements() == 1',
            '$_->get_exported_name() =~ /A/',
        ],
}

Probe : {
      name => "RESPONSE",
      classes => ComplexInstance,
      filters => [
            '$_->get_exported_name() =~ /A.*Y/',
        ],
}

Probe : {
      structure => X,
}
Probe : {
      structure => Y,
}

#-----------------------------------------------------
# INITIAL CONDITIONS
#-----------------------------------------------------
# give non-reference state a non-zero IC
Init : {
      structure => A,
      state => '[T,x,x]',
      IC=> 1.0,
}
Init : {
      structure => X,
      IC => 0.0,
}
Init : {
      structure => Y,
      IC => 1.0,
}

#-----------------------------------------------------
# STIMULUS
#-----------------------------------------------------
```

```
# Clamp X at successively different levels and bring
# to steady-state each time. In matlab, the variable
# event_times will give the time at which steady-state
# was reached.
Stimulus : {
      structure => 'X',
      type => "dose_response",
      strength => 1000,
      range => [1e-3,1e3],
      steps => 12,
      log_steps => 1,
}

################################
CONFIG:
################################
t_final = 100000
t_vector = [0:1:tf]

matlab_ode_solver = ode15s
```

3 Supplementary References

1. Jackson MB (2006) Molecular and cellular biophysics. Cambridge ; New York: Cambridge University Press. xiii, 512 p. p.
2. Leffler JE (1953) Parameters for the Description of Transition States. Science 117: 340-341.
3. Stefan MI, Edelstein SJ, Le Novere N (2008) An allosteric model of calmodulin explains differential activation of PP2B and CaMKII. Proc Natl Acad Sci U S A 105: 10768-10773.
4. Schlessinger J (2002) Ligand-induced, receptor-mediated dimerization and activation of EGF receptor. Cell 110: 669-672.
5. Ozcan F, Klein P, Lemmon MA, Lax I, Schlessinger J (2006) On the nature of low- and high-affinity EGF receptors on living cells. Proc Natl Acad Sci U S A 103: 5735-5740.
6. Asakura S, Honda H (1984) Two-state model for bacterial chemoreceptor proteins. The role of multiple methylation. J Mol Biol 176: 349-367.
7. Barkai N, Leibler S (1997) Robustness in simple biochemical networks. Nature 387: 913-917.
8. Sourjik V, Berg HC (2004) Functional interactions between receptors in bacterial chemotaxis. Nature 428: 437-441.
9. Monod J, Wyman J, Changeux JP (1965) On the Nature of Allosteric Transitions: A Plausible Model. J Mol Biol 12: 88-118.
